# Gut microbiome-shaped chemotherapy responses across cancer types: mechanisms of resistance, biomarkers, and targeted interventions

**DOI:** 10.3389/fimmu.2026.1945287

**Published:** 2026-10-05

**Authors:** Ting Shao, Minghui Li, Shaolin Li, Jie Pu, Chunsheng Li, Changgang Guo

**Affiliations:** 1 Department of Gynecology, Affiliated Hospital of Chifeng University, Chifeng, China; 2 Department of Urology, Affiliated Hospital of Chifeng University, Chifeng, China; 3 General Office, Affiliated Hospital of Chifeng University, Chifeng, China

**Keywords:** biomarkers, chemoresistance, gut microbiome, microbial metabolites, microbiome-targeted therapy

## Abstract

Cytotoxic chemotherapy remains an important component of multimodal treatment for many solid tumors, but resistance and dose-limiting toxicities continue to constrain clinical benefit. The gut microbiome can influence chemotherapy response through microbial drug biotransformation, metabolite signaling, intestinal-barrier integrity, systemic immune regulation, and tumor-cell stress and death pathways. Across solid tumors, the evidence is strongest in colorectal cancer, where clinical associations are supported by increasingly detailed mechanistic and interventional studies; evidence in many extraintestinal cancers remains less mature and is dominated by smaller observational cohorts and selected preclinical models. Importantly, gut-derived systemic effects should be distinguished from local or tumor-associated microbial mechanisms, and direct tumor-cell resistance should be separated from altered drug exposure and toxicity-mediated reductions in relative dose intensity. Microbiota-associated and metabolic biomarkers and microbiota-targeted interventions show potential for treatment stratification, toxicity prediction, and therapeutic modulation, but clinical translation remains limited by heterogeneous cohorts, incomplete causal validation, variable engraftment and strain specificity, and safety and standardization requirements. Overall, the microbiome is best viewed as a cancer- and regimen-specific modifier of chemotherapy rather than a universal determinant of response. Prospective, mechanism-matched studies are needed to establish whether microbiome-guided strategies can improve treatment efficacy, tolerance, and completion.

## 1 Introduction

The gut microbiota comprises microbial communities, including bacteria, fungi, archaea, and viruses, that colonize the gastrointestinal tract; the gut microbiome further encompasses the genes, functional activities, metabolites, and ecological environment of these microorganisms. The gut microbiome participates in the utilization of dietary substrates, vitamin and bile acid metabolism, maintenance of the mucosal barrier, immune maturation, and xenobiotic transformation. Microbial metabolites, cell-wall components, and immune signals can also enter the circulation and affect the liver, lungs, kidneys, bone marrow, distant mucosal sites, and tumor tissues, providing a physiological basis for gut microbiome-mediated regulation of the biology and drug responses of extraintestinal tumors (1, 2).

Despite the expanding use of targeted therapies and immunotherapies, platinum agents, fluoropyrimidines, taxanes, anthracyclines, topoisomerase inhibitors, and alkylating agents remain widely used in neoadjuvant, adjuvant, and advanced treatment of a variety of solid tumors (3, 4). Their clinical effects are constrained by primary resistance, acquired resistance that develops under treatment pressure, and host treatment tolerance. Altered drug uptake or efflux, metabolic reprogramming of drug metabolism, enhanced DNA damage repair, evasion of cell death, cancer stemness, and metabolic adaptation can directly reduce tumor-cell sensitivity; diarrhea, nausea and vomiting, infection, myelosuppression, and hepatic and renal injury mainly reduce relative dose intensity through dose reduction, delay, or interruption and should not be equated with tumor-cell chemoresistance (5, 6). Direct tumor-cell resistance, microbiota-mediated changes in drug exposure or biotransformation, and toxicity-mediated reductions in treatment exposure or relative dose intensity therefore represent distinct dimensions of chemotherapy response. Conventional research on drug resistance has mainly focused on tumor genomes and intracellular signaling networks, whereas microbe-derived drug metabolism and immune and inflammatory signals indicate that the host microecology constitutes another layer of treatment-response regulation (5).

Microbial communities can alter chemotherapy response through compartment-specific drug biotransformation and host regulation. Within the intestinal compartment, strain-specific microbial enzymes can chemically modify drugs and alter host drug disposition; for example, bacterial β-glucuronidase can reconvert the irinotecan metabolite SN-38G into active SN-38 and exacerbate delayed diarrhea (7, 8). A distinct local mechanism operates within tumors, where intratumoral bacteria expressing the long isoform of cytidine deaminase can inactivate gemcitabine and reduce local drug activity in experimental models (9). Short-chain fatty acids (SCFAs), secondary bile acids, tryptophan derivatives, and polyamines can also regulate epigenetics, redox homeostasis, and cell death; barrier injury and translocation of microbe-associated molecules can activate inflammatory and survival pathways and remodel myeloid and lymphocyte responses (4, 5).

The gut microbiome and chemotherapy have a dynamic bidirectional relationship. Longitudinal patient studies show that chemotherapy can remodel microbial composition and metabolic function, although the direction of change is influenced by the drug, cancer type, and supportive care (3, 10). In germ-free or antibiotic-treated mice, oxaliplatin-induced reactive oxygen species (ROS) generation by tumor-infiltrating myeloid cells and tumor killing are impaired, providing causal evidence for the involvement of commensal bacteria in platinum-drug efficacy (11); increased mucosal permeability and translocation of specific commensals caused by cyclophosphamide can induce helper T cell responses that contribute to its antitumor effects (12). Baseline microbiota may affect initial sensitivity, whereas ecological selection and mucosal injury caused by chemotherapy can alter drug metabolism, systemic immunity, and organ toxicity during subsequent cycles. Microbial changes associated with treatment should not be uniformly regarded as harmful dysbiosis; their net effect depends on the drug, strain function, sampling time, and host background (4, 5).

The available evidence is clearly cancer- and regimen-dependent. Among the cancer types reviewed here, colorectal cancer (CRC) currently has the most developed evidence base, spanning fecal and tumor-associated microorganisms, microbial metabolites, drug biotransformation, patient cohorts, and mechanistic intervention studies; in contrast, evidence in lung, breast, gynecological, and genitourinary cancers remains less extensive and is dominated by smaller clinical cohorts and selected preclinical models (3, 13). Systematic reviews indicate that most human studies are small observational studies, with differences in the direction of candidate taxa, control of confounders, and sequencing workflows (3). Differential taxa in patient cohorts are therefore better regarded as candidate predictive clues than as direct explanations for resistance; transferable biomarkers are also more likely to comprise strains, functional genes, metabolic pathways, immune indicators, and community stability rather than a single genus or diversity index (1, 3). Most cancer types have not yet formed a complete evidence loop from patient association and animal causality through cellular mechanisms to clinical intervention.

This review integrates clinical associations and experimental mechanisms linking the gut microbiome and cytotoxic-chemotherapy response from a cross-cancer perspective. It places microbial composition, metabolites, drug biotransformation, the mucosal barrier, systemic immunity, the tumor microenvironment (TME), and tumor-cell death and stress adaptation within a single framework, and compares shared mechanisms and specificities across cancer types, chemotherapy regimens, and microbial niches. On this basis, it evaluates the value of multi-omics indicators for pretreatment stratification, dynamic monitoring, and toxicity warning, and examines the translational boundaries of microbiota-targeted interventions, including fecal microbiota transplantation (FMT), probiotics or postbiotics, and bacteria-directed phages, together with emerging microbe-inspired adjunctive technologies such as engineered microorganisms and bacteria-derived delivery platforms. Its clinical objective is not to broadly increase microbial diversity, but to identify patients at risk of ineffective chemotherapy or high toxicity, restore regimen-matched drug sensitivity, and maintain relative dose intensity by reducing dose-limiting toxicities; achieving these aims still depends on longitudinal causal validation, standardization, risk stratification, and mechanism-matched clinical endpoints. Mechanistically, three distinct categories are relevant to microbiome–chemotherapy interactions. Gut-derived systemic mechanisms originate in the intestinal microbial community and influence chemotherapy through circulating metabolites or microbial products, mucosal-barrier disruption, systemic immune remodeling, or altered host drug disposition. Local microbial mechanisms arise from microorganisms or microbial enzymes within tumor tissue or other organ-specific niches and can directly modify drug activity, tumor metabolism, or tumor-cell survival. Microbe-inspired therapeutic technologies, by contrast, exploit microbial structures, tropism, or synthetic-biology principles for drug delivery, immune modulation, stromal remodeling, or combination treatment. Cross-cancer or cross-compartment evidence provides transferable mechanistic frameworks for interpreting microbiome–chemotherapy interactions, while direct validation in the relevant tumor type or microbial niche determines cancer- and compartment-specific applicability. Metabolite-mediated effects can likewise be interpreted across distinct evidence levels, including microbiota-dependent *in vivo* effects, microbiota-associated metabolite changes, and pharmacological effects demonstrated by exogenous compound administration; the latter establish compound-level biological activity whose physiological relevance depends on experimental exposure relative to concentrations achieved in humans.

## 2 Regulatory roles of the gut microbiome in colorectal cancer chemotherapy response

Chemoresistance in CRC is a major clinical challenge in oncology worldwide. Its molecular mechanisms have long been considered to arise mainly from intrinsic genomic variation and signaling-pathway abnormalities in tumor cells. However, CRC spans anatomically contiguous luminal, mucosal, and tumor-associated microbial compartments, and evidence relevant to chemotherapy response therefore includes both intestinal-community effects and local tumor-associated mechanisms. Gut-derived effects operate through intestinal microbial composition, metabolites, barrier function, and systemic immunity, whereas tumor-associated microorganisms can interact directly with cancer cells or the local microenvironment. Within CRC, these spatially distinct microbial influences converge on three interconnected levels of chemotherapy response: direct modulation of tumor-cell sensitivity through cell-death pathways, immune evasion, transcriptional regulation, and drug efflux; metabolite-mediated regulation of tumor and immune responses; and chemotherapy-induced microbial remodeling with feedback on drug activity, treatment toxicity, and treatment completion (**Table 1**).

**Table 1:** Microbiota-associated mechanisms and biomarkers of chemotherapy response and toxicity in colorectal cancer.

Microbial factor	Study population/experimental model	Core mechanism	Chemotherapy-related finding	Reference
*F. nucleatum*	80 human colon cancer samples; colon cancer cell lines co-cultured with *F. nucleatum*	*F. nucleatum* infection upregulates PVT1 via TLR4/NF-κB pathway; PVT1 binds to ATAD3A and prevents its ubiquitination, inhibiting ER stress-mediated cell death	Promotes oxaliplatin resistance; *F. nucleatum* abundance correlates with later clinical stage and shorter relapse-free survival	(14)
	CRC xenografts, ApcMin/+ mice, AOM/DSS-induced CRC mice	*F. nucleatum* regulates Hippo pathway (LATS1/2–YAP/TAZ) to promote BCL2 expression, inhibiting caspase-3/GSDME-mediated pyroptosis	Promotes chemoresistance; *F. nucleatum* enrichment in recurrent CRC patients correlates with poor prognosis	(15)
	CRC cell lines; CRC mouse models	*F. nucleatum* activates E-cadherin/β-catenin/TCF4 axis to promote GPX4 overexpression, inhibiting ferroptosis	Promotes oxaliplatin resistance	(16)
	*In vitro* co-culture transcriptomic datasets (meta-analysis)	*F. nucleatum* activates inflammation-driven, immune evasion, DNA damage, and metastasis pathways; induces type I and II interferon signaling to mimic “pseudo-antiviral state”	Promotes chemoresistance through transcriptional reprogramming	(17)
	Stage III/IV CRC patients	*F. nucleatum* abundance in tumor tissues associated with tumor invasion, lymph node metastasis, and distant metastasis; *F. nucleatum* level distinguishes adjuvant chemotherapy benefit	Predictive biomarker; low *F. nucleatum* patients derive more benefit from adjuvant chemotherapy; *F. nucleatum* is an independent poor prognostic factor	(18)
*B. fragilis*	Two independent CRC patient cohorts; CRC xenografts; ApcMin/+ mice; AOM/DSS-induced CRC mice	*B. fragilis* surface protein SusD/RagB binds to host Notch1 receptor; activates Notch1 signaling; induces EMT and cancer stem cell features; suppresses 5-FU and oxaliplatin-induced apoptosis	Promotes chemoresistance; *B. fragilis* abundance correlates with poor survival in nonresponders	(19)
pks^+^ *E. coli* (CoPEC)	CRC cell lines; CRC mouse models	CoPEC produces colibactin via pks gene island; induces DNA double-strand breaks; promotes phosphatidylcholine remodeling via Land’s cycle; accumulates lipid droplets in infected cancer cells to maintain survival under chemotherapeutic stress	Promotes chemoresistance; CoPEC colonization correlates with lower overall survival in stage III-IV CRC patients	(20)
*E. coli* (preTA^+^)	Germ-free and antibiotic-treated mice; CRC patient fecal samples	preTA operon-encoding bacteria convert active 5-FU to inactive metabolites; preTA^+^ *E. coli* transplantation rescues fluoropyrimidine toxicity	Reduces local 5-FU activity through microbial drug inactivation; preTA abundance negatively correlates with fluoropyrimidine toxicity	(21)
*P. anaerobius*	Chemoresistant and chemosensitive CRC patients; MC-38 mouse model	*P. anaerobius* recruits MDSCs into tumor microenvironment; MDSCs secrete IL-23 to activate STAT3–EMT pathway; promotes IL-23 upregulation in tumor tissues	Promotes oxaliplatin resistance; *P. anaerobius* abundance correlates with IL-23 levels and chemoresistance	(22)
	MC-38 mouse model	*P. anaerobius* recruits MDSCs; MDSCs secrete IL-23 to activate STAT3–EMT pathway	Promotes oxaliplatin resistance	(23)
*D. desulfuricans*	CRC patient cohort; syngeneic transplantation mouse model	*D. desulfuricans* elevates serum S-adenosylmethionine (SAM); SAM drives METTL3 expression, reducing CRC cell sensitivity to FOLFOX	Promotes FOLFOX resistance; high *Desulfovibrio* abundance indicates poor neoadjuvant FOLFOX outcomes	(24)
*K. pneumoniae*	CRC cell lines; LPS-stimulated macrophages/fibroblasts	*K. pneumoniae* LPS activates TLR4–NF-κB pathway; suppresses RNA-binding factor Wig-1, destabilizing TP53 mRNA; macrophage-derived extracellular vesicles mediate p53 degradation in cancer cells	Promotes chemoresistance via paracrine p53 suppression; LPS-induced inflammation correlates with reduced survival following chemotherapy	(25)
*E. faecalis*	CRC tumor tissue analysis	*E. faecalis* enrichment in CRC tumor tissues is associated with PI3K/AKT/mTOR pathway activation and VEGFA-related angiogenic signaling.	Associated with CRC progression and chemoresistance-related signaling; vancomycin-resistant *E. faecalis* shows stronger PI3K/AKT/mTOR activation, although direct effects on chemotherapy sensitivity remain to be established.	(26)
*S. gallolyticus*	CRC-associated clinical isolates	*S. gallolyticus* induces NF-κB/IL-8 signaling and upregulates anti-apoptotic programs.	NF-κB/IL-8 and anti-apoptotic signaling may reduce chemotherapy-induced cell death; direct clinical evidence of chemoresistance remains limited.	(27)
Multiple bacteria (consortium)	CRC patients	Six genera (*Roseburia, Helicobacter, Gardnerella, Flavonifractor, Coprococcus, Anaerostipes*) associated with relapse-free survival; *Roseburia* and Fusobacteria enriched in resistant group; *Bifidobacterium*, *Helicobacter*, *Akkermansia* enriched in non-resistant group	Predictive biomarker; intratumor microbiome features and circulating microbial DNA can predict CRC resistance	(28)
*A. muciniphila*	CRC patient cohort; mouse models	*A. muciniphila* activates tryptophan metabolism, elevates 5-hydroxytryptophan; promotes intratumoral CD8^+^ T cell infiltration and interferon γ (IFN-γ) upregulation; *A. muciniphila* components (Amuc_2172, Amuc_1434) promote M1-like macrophage polarization via TLR2/NLRP3 signaling	Predicts chemotherapy sensitivity; fecal *A. muciniphila* abundance positively correlates with chemotherapy responsiveness	(29)
*A. muciniphila*/*F. prausnitzii*	Advanced CRC patients	S100A12 and sCD14 levels positively correlate with *F. nucleatum* and *Prevotella* abundance; negatively correlate with *F. prausnitzii* and *A. muciniphila*; nonresponders have higher baseline S100A12 and sCD14	Predictive biomarker; combined S100A12 + sCD14 predicts chemoresistance (AUC = 0.838); these markers inversely correlate with chemosensitivity-associated bacteria	(30)
*Lactobacillus* (genus-level enrichment)	mCRC patients receiving first-line chemotherapy	Chemotherapy responders harbor more beneficial bacteria, notably *Lactobacillus*; *Bifidobacterium* abundance increased in non-progressive disease group; β-diversity differs by RAS mutation status (*Holdemanella, Anaerostipes, Collinsella* enriched in RAS-mutant patients)	Predictive biomarker; *Lactobacillus* enrichment correlates with chemotherapy response; *Bifidobacterium* correlates with non-progressive disease	(31)
*Bifidobacterium* (genus-level depletion)	CRC patients; AOM/DSS mouse model	*Bifidobacterium* depletion in CRC patients associated with *F. nucleatum* enrichment and lipid metabolite accumulation; *B. animalis* inhibits *F. nucleatum* and CRC cell growth in an acid-dependent manner	Predicts chemoresistance; *Bifidobacterium* depletion correlates with *F. nucleatum*-driven chemoresistance; *B. animalis* supplementation may alleviate *F. nucleatum*-induced lipid accumulation and tumor growth	(32)
	AOM/DSS-induced CRC mouse model	FMT from healthy mice reverses gut dysbiosis; increases CD8^+^ T and CD49b^+^ NK cell infiltration; reduces immunosuppressive Foxp3^+^ Treg cells; downregulates IL1a, IL6, IL12a, IL12b, IL17a; upregulates IL10	Predicts chemotherapy sensitivity; FMT restores *Bifidobacterium* and other beneficial genera; enhances antitumor immune responses	(33)

CRC, colorectal cancer; mCRC, metastatic colorectal cancer; F. nucleatum, Fusobacterium nucleatum; B. fragilis, Bacteroides fragilis; E. coli, Escherichia coli; P. anaerobius, Peptostreptococcus anaerobius; D. desulfuricans, Desulfovibrio desulfuricans; K. pneumoniae, Klebsiella pneumoniae; E. faecalis, Enterococcus faecalis; S. gallolyticus, Streptococcus gallolyticus; A. muciniphila, Akkermansia muciniphila; F. prausnitzii, Faecalibacterium prausnitzii; E. dolichum, Eubacterium dolichum; PVT1, plasmacytoma variant translocation 1; TLR4, Toll-like receptor 4; NF-κB, nuclear factor kappa-light-chain-enhancer of activated B cells; ATAD3A, ATPase family AAA domain-containing protein 3A; ER, endoplasmic reticulum; YAP, Yes-associated protein; TAZ, transcriptional coactivator with PDZ-binding motif; BCL2, B-cell lymphoma 2; GSDME, gasdermin E; EMT, epithelial–mesenchymal transition; GPX4, glutathione peroxidase 4; AOM/DSS, azoxymethane/dextran sodium sulfate; CoPEC, colibactin-producing Escherichia coli; MDSC, myeloid-derived suppressor cell; STAT3, signal transducer and activator of transcription 3; SAM, S-adenosylmethionine; METTL3, methyltransferase-like protein 3; OXA, oxaliplatin; FOLFOX, folinic acid, 5-fluorouracil, and oxaliplatin; XELOX, capecitabine + oxaliplatin; 5-FU, 5-fluorouracil; S100A12, S100 calcium-binding protein A12; sCD14, soluble cluster of differentiation 14; sRAGE, soluble receptor for advanced glycation end products; ROC, receiver operating characteristic; AUC, area under the curve; CSCs, cancer stem cells; preTA, fluoropyrimidine preTA operon; IFN-γ, interferon γ; TLR, Toll-like receptor; NLRP3, NLR family pyrin domain containing 3; cGAS–STING, cyclic GMP–AMP synthase–stimulator of interferon genes; VREs, vancomycin-resistant enterococci; PI3K, phosphoinositide 3-kinase; AKT, protein kinase B; mTOR, mammalian target of rapamycin; VEGFA, vascular endothelial growth factor A.

### 2.1 Direct regulation of CRC chemotherapy sensitivity by changes in microbial abundance

Enrichment of *F. nucleatum* is the most extensively studied pro-resistance marker in CRC and is closely associated with poor prognosis. It synergistically drives chemoresistance through multiple mechanisms, including inhibition of several forms of cell death: (a) it promotes BCL2 expression by regulating the Hippo signaling pathway (LATS1/2-YAP/TAZ) and specifically inhibits caspase-3/GSDME-mediated pyroptosis (15); (b) it promotes GPX4 overexpression through the E-cadherin/β-catenin/TCF4 transcriptional axis, inhibits ferroptosis, and thereby induces oxaliplatin resistance (16); (c) it upregulates lncRNA PVT1 through the TLR4/NF-κB pathway, and PVT1 binds ATAD3A and prevents its ubiquitin-mediated degradation, inhibits cell death mediated by endoplasmic reticulum stress, and enhances oxaliplatin resistance (14); (d) its FadA adhesin binds E-cadherin to activate β-catenin signaling, while Fap2 binds TIGIT to inhibit natural killer (NK) cell and T cell cytotoxicity. These two pathways promote enrichment of myeloid-derived suppressor cells (MDSCs) and regulatory T (Treg) cells in the TME and maintain tumor-cell survival through a ULK1-dependent autophagy pathway (34); and (e) extracellular intratumoral *Fusobacterium* disrupts E-cadherin-mediated intercellular epithelial contacts, induces G_0_/G_1_ phase arrest and transcriptional quiescence, and induces resistance to 5-fluorouracil (5-FU) (35). In addition, a meta-analysis incorporating multiple independent coculture experiments showed that *F. nucleatum* activates multiple chemoresistance-related pathways involving inflammation, immune escape, DNA damage, and metastasis, for example, by inducing activation of type I and type II interferon signaling and simulating a pseudo-antiviral state through transcriptional reprogramming to weaken drug efficacy (17). At the transcriptional level, *F. nucleatum* drives chemoresistance by regulating expression of multiple noncoding RNAs, including regulation of miR-1246 and miR-135b to affect the Wnt/β-catenin pathway, miR-22 and miR-135b to affect the PI3K/AKT pathway, and miR-31 and lnc-NEAT1 to affect the TLR4/NF-κB pathway (36). A more direct mechanism of resistance is that *F. nucleatum* can promote oxaliplatin resistance by upregulating the drug-efflux transporter ABCG2 through E-cadherin/β-catenin signaling (37).

In addition to *F. nucleatum*, *Bacteroides fragilis* is also significantly enriched in patients with chemoresistant CRC, and two independent cohorts have confirmed that its abundance is associated with poor survival. Mechanistically, *B. fragilis* physically adheres to CRC cells; its surface proteins SusD/RagB directly bind the host Notch1 receptor, activate Notch1 signaling and NICD nuclear translocation, and induce epithelial–mesenchymal transition (EMT) and tumor-cell stemness, thereby inhibiting apoptosis induced by 5-FU and oxaliplatin. Loss of SusD/RagB, blockade of Notch1 signaling, and targeted clearance by phage VA7 can each abolish *B. fragilis*-mediated chemoresistance (19). In addition, colibactin-producing *Escherichia coli* (CoPEC) produces the genotoxin colibactin through its pks genomic island, inducing DNA double-strand breaks and interstrand crosslinks in host intestinal epithelial cells. More importantly, CoPEC promotes phosphatidylcholine remodeling through colibactin, causing lipid-droplet accumulation in infected CRC cells to maintain survival under chemotherapy stress; this metabolic reprogramming can be reversed by acyl-CoA synthetase inhibitors (20). *Peptostreptococcus anaerobius promotes oxaliplatin resistance in experimental models by recruiting MDSCs into the tumor microenvironment, where MDSC-derived IL-23 activates the STAT3–EMT pathway* (23)*. Consistent with this mechanism*, clinical data showed that fecal *P. anaerobius* abundance was higher in chemoresistant patients and positively correlated with tumor-tissue IL-23 levels (22). After colonization of the intestine, *Desulfovibrio desulfuricans* can increase serum S-adenosylmethionine (SAM) levels, and SAM reduces CRC-cell sensitivity to folinic acid, 5-fluorouracil, and oxaliplatin (FOLFOX) chemotherapy by driving expression of methyltransferase-like protein 3 (METTL3) (24).

In contrast to pro-resistance bacteria, *Akkermansia muciniphila* and related bacteria are positively associated with chemotherapy sensitivity. Clinical observations indicate that fecal *A. muciniphila* abundance is positively correlated with chemotherapy responsiveness. Huanglian Jiedu Decoction (HLJDD) selectively promotes *A. muciniphila* proliferation, enriches tryptophan metabolism, and increases 5-hydroxytryptophan levels, leading to intratumoral CD8^+^ T cell infiltration and interferon γ (IFN-γ) upregulation and thereby enhancing chemotherapy efficacy (29). Further studies indicate that nonviable forms of *A. muciniphila*, including pasteurized bacteria, outer membrane vesicles (OMVs), and purified proteins (Amuc_1100, Amuc_2172, and Amuc_1434), promote M1-like macrophage polarization and activate CD8^+^ T cells through the TLR2/NLRP3 signaling pathway, sensitizing tumors to chemotherapy and programmed cell death protein 1 (PD-1) blockade. However, the effects of live *A. muciniphila* are clearly context-dependent; in inflammatory settings, such as colitis-associated CRC, or after antibiotic exposure, it may exert tumor-promoting effects by excessive mucus degradation and disruption of the intestinal barrier (38). In addition, *Lactobacillus* spp. restore chemotherapy sensitivity through several mechanisms: (a) extracellular vesicles derived from *Lactobacillus plantarum* (LpEVs) reverse metabolic reprogramming in 5-FU-resistant CRC cells through a PDK2-mediated glucose-metabolism pathway, inhibit proliferation, and induce apoptosis (39); (b) γ-aminobutyric acid (GABA)-producing *L. plantarum* activates antiproliferative and antimigratory effects through GABA_B_ receptor signaling and induces apoptosis in 5-FU-resistant cells (40); and (c) combined treatment with *L. plantarum* supernatant and 5-FU reverses resistance and reduces colonosphere formation by inactivating Wnt/β-catenin signaling (41). *Bifidobacterium* spp. induce oxidative stress and mitochondrial dysfunction in CRC cells by inhibiting ALB gene expression, and their supernatant can also inhibit colon cancer cell progression by interfering with TGF-β signaling and suppressing CEA gene expression (42, 43). Notably, synergistic or antagonistic effects may occur among different microbiota: the T helper 17 (Th17) inflammatory microenvironment induced by enterotoxigenic *B. fragilis* (ETBF) may create favorable conditions for CoPEC colonization and colibactin exposure, whereas colibactin-induced changes in DNA repair status may in turn affect resistance pathways driven by other microbiota. Such complex interspecies interactions make functional analysis of a single taxon insufficient to fully reflect the *in vivo* situation (44, 45) (**Figure 1**; **Table 1**).

**Figure 1:**
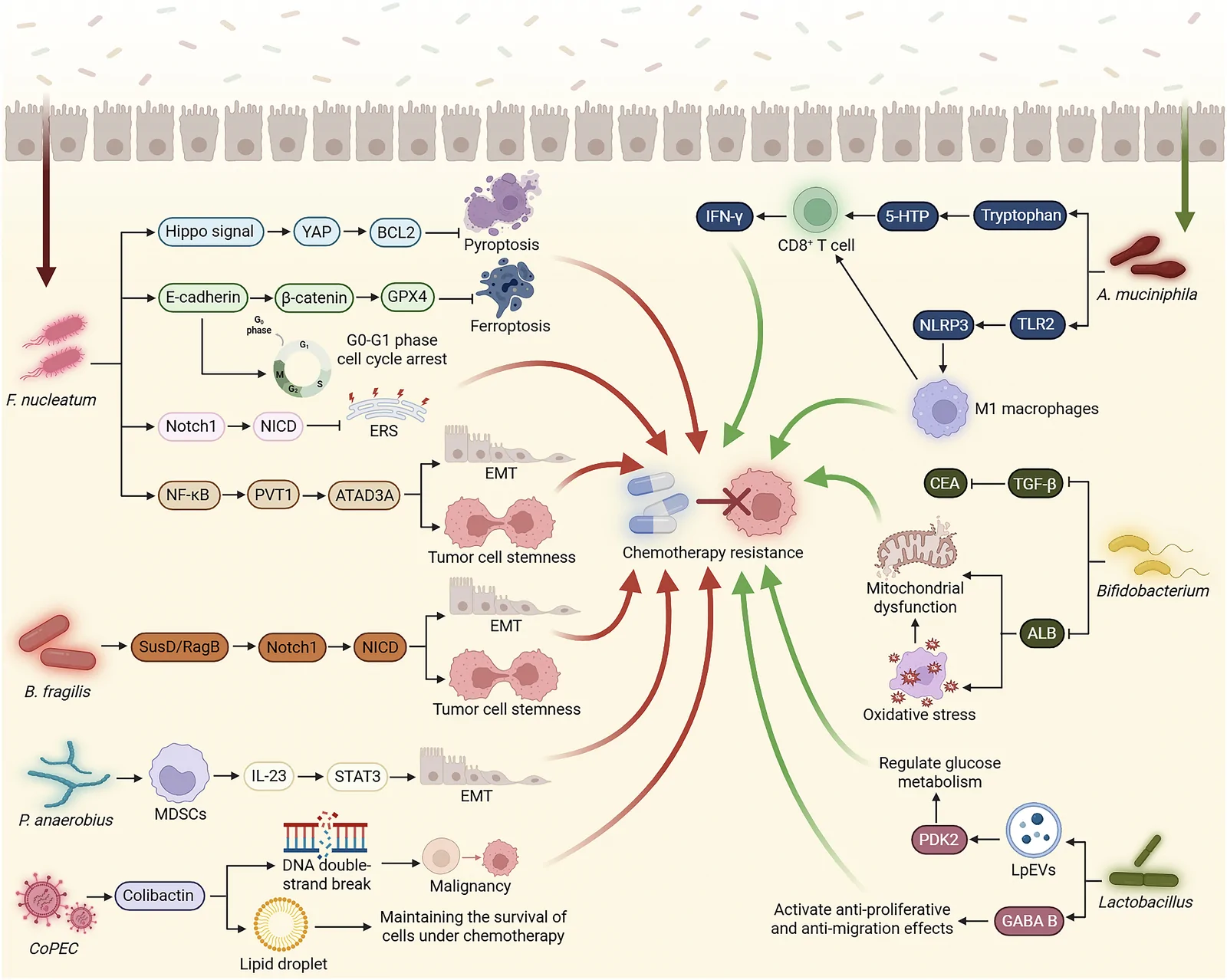
Intestinal and tumor-associated bacteria modulate chemotherapy resistance and sensitivity in colorectal cancer. The figure summarizes microbial mechanisms that influence chemotherapy response through tumor-cell death, epithelial–mesenchymal transition and cancer stemness, immune regulation, DNA damage, and metabolic adaptation. Fusobacterium nucleatum, Bacteroides fragilis, Peptostreptococcus anaerobius, and colibactin-producing Escherichia coli illustrate representative resistance-associated mechanisms, whereas Akkermansia muciniphila, Bifidobacterium, and Lactobacillus illustrate mechanisms associated with enhanced chemotherapy sensitivity. The pathways integrate mechanistic evidence from cellular and animal models with selected clinical associations; study-specific evidence is summarized in **Table 1**. Arrowheads indicate promotion or activation, whereas blunt-ended lines indicate inhibition. Red lines indicate effects that promote chemotherapy resistance or reduce chemotherapy sensitivity, whereas green lines indicate effects that inhibit chemotherapy resistance or enhance chemotherapy sensitivity. EMT, epithelial–mesenchymal transition; MDSC, myeloid-derived suppressor cell; 5-HTP, 5-hydroxytryptophan; LpEVs, extracellular vesicles derived from Lactobacillus plantarum.

### 2.2 Gut microbial metabolites and CRC chemotherapy response

Microbe-derived metabolites, including SCFAs, secondary bile acids, polyamines, and tryptophan derivatives, extensively reshape the CRC metabolic network and regulate the immune microenvironment, thereby affecting tumor progression and treatment response (46).

SCFAs play a dual role in the regulation of chemoresistance: they can reverse resistance through epigenetic modification but can also induce resistance by activating proliferative signaling pathways. The core antitumor mechanism of butyrate is inhibition of histone deacetylase (HDAC) activity, which increases histone H3/H4 acetylation and activates silenced tumor-suppressor genes (47). In addition, butyrate induces G_2_/M phase arrest and apoptosis by inhibiting thymidylate synthase (TYMS) expression, enhances CRC-cell sensitivity to 5-FU (48), and suppresses tumor-tissue colonization and invasion by *F. nucleatum* by downregulating its adhesion-associated outer-membrane proteins RadD, FomA, and FadA, thereby alleviating oxaliplatin resistance (49). Microbe-derived butyrate can also promote chemoresistance: butyrate produced by *Roseburia* and *Fusobacteria* enhances CRC-cell proliferation and migration through activation of PI3K–AKT signaling, increases the IC_50_ values of 5-FU and docetaxel, and results in resistance (28). During this process, CRC cells can evade the effects of butyrate through differential expression of HDAC isoenzymes, downregulation of the butyrate transporter SMCT1, and metabolic reprogramming, leading to resistance to butyrate and its chemotherapy-sensitizing effects (50). Propionate, as a distinct acyl-lysine histone mark (H3K18pr, H3K18bu, H4K12pr, and H4K12bu), directly modifies specific genomic regions and increases chromatin accessibility. Sodium propionate also enhances CRC-cell sensitivity to 5-FU by inhibiting thymidylate synthase expression (51). Metabolites derived from *L. plantarum* can restore functional SMCT1 expression through epigenetic or transcriptional regulation, resensitizing 5-FU-resistant CRC cells to 5-FU (52). Several of the SCFA mechanisms described above were established using exogenously administered butyrate or propionate rather than by directly manipulating microbial metabolite production *in vivo*. These experiments establish the capacity of SCFAs to modify tumor-cell responses to chemotherapy and define compound-level mechanisms whose microbiome-level relevance depends on whether comparable tumor exposures are achieved through endogenous microbial production *in vivo*. Notably, the pharmacologic effects of SCFAs are cell-type-dependent: in microsatellite instability (MSI) CRC cells, SCFA-induced DNA damage more effectively triggers upregulation of chemokines, major histocompatibility complex class I (MHC I), and antigen-processing/presentation genes and activates CD8^+^ T cell immunity, whereas this effect is markedly weaker in chromosomally unstable (CIN) cells (53).

These apparently opposing effects can be reconciled through an exposure- and context-dependent framework. First, extracellular concentration and exposure duration determine the amount of butyrate available for cellular uptake, but fecal abundance cannot be equated with portal, circulating, or intratumoral exposure. Second, SMCT1 expression, mitochondrial oxidation, metabolic reprogramming, and the HDAC isoenzyme profile determine whether intracellular butyrate primarily acts as a metabolic substrate or accumulates sufficiently to inhibit HDAC activity. Third, CRC molecular subtype modifies the downstream response. MSI cells can mount stronger DNA-damage, antigen-presentation, and CD8^+^ T cell programs after SCFA exposure, whereas CIN cells show a weaker immunogenic response. Finally, the producing strain and surrounding microbial community influence co-metabolites, local nutrient conditions, and inflammatory signaling. These variables may favor HDAC inhibition and TYMS downregulation in some models but PI3K–AKT activation and resistance in others. Interpretation across studies therefore depends on the microbial source, butyrate concentration and exposure duration, CRC subtype, and transporter status, as well as on whether experimentally active exposures overlap with physiological levels in humans. These context-dependent differences may account for the apparently divergent sensitizing and resistance-promoting effects of butyrate across experimental systems.

Some intestinal 7α-dehydroxylating bacteria, such as *Clostridium scindens*, can convert primary bile acids into deoxycholic acid (DCA) and lithocholic acid (LCA); a high-fat, high-protein Western diet can promote this process. Secondary bile acids in the colon can drive tumorigenesis and resistance through DNA damage, oxidative stress, and Wnt/β-catenin activation (54, 55). DCA induces mitochondrial oxidative stress through multiple pathways involving NADPH oxidase, Na^+^/K^+^-ATPase, cytochrome P450, calcium ions, and mitochondrial respiratory-chain complex IV; it activates NF-κB and initiates antiapoptotic survival pathways, forming an important molecular basis for CRC chemoresistance driven by secondary bile acids. LCA can drive ROS generation by activating the Src/EGFR signaling axis and subsequently phosphorylate and activate ERK1/2 and AKT, which upregulate IL-8 expression through activator protein 1 (AP-1) and NF-κB, respectively. IL-8 is not only a proangiogenic factor but has also been shown to induce CRC-cell chemoresistance to 5-FU and oxaliplatin. After piperine inhibits Src/EGFR activity, it can block the downstream ERK1/2/AP-1 and AKT/NF-κB signaling cascades and effectively inhibit LCA-induced IL-8 expression (56). miR-21 is one of the most extensively studied oncogenic miRNAs in CRC; its high expression is closely associated with poor prognosis and chemoresistance. LCA also synergistically upregulates miR-21 transcription by inhibiting STAT3 signaling and activating the ERK1/2–AP-1 transcription factor. Increased miR-21 subsequently targets and inhibits the tumor-suppressor gene PTEN, relieving inhibition of the PI3K/AKT signaling pathway and ultimately promoting CRC-cell proliferation (57). DCA and LCA can inhibit Wnt/β-catenin signaling, downregulate P-glycoprotein expression, and increase 5-FU exposure in intestinal epithelial cells; excessive DCA and LCA can exacerbate capecitabine-induced diarrhea (58). Secondary bile acids have only weak effects on wild-type p53 but markedly promote aggregation of mutant p53 in CRC cell lines carrying R273H and R273C mutations; this effect is substantially enhanced in the presence of doxorubicin, suggesting that aggregation induced by bile acids and phase separation of mutant p53, together with sequestration of doxorubicin, may contribute to chemoresistance (59).

Importantly, evidence that gut bacteria generate DCA and LCA and evidence for their direct effects on tumor-cell signaling derive from different experimental settings. These studies establish biologically plausible DCA- and LCA-responsive tumor-cell pathways, although the microbiome-level relevance of these mechanisms depends on the concentrations achieved through endogenous microbial production *in vivo*.

Microbe-derived polyamine metabolites also play important regulatory roles in CRC chemoresistance, with clearly species-dependent effects. Spermidine concentrations are significantly increased in culture supernatants from CRC-associated pathogenic microbiota, including ETEC, *Peptostreptococcus anaerobius*, and *F. nucleatum* (60). Tumor biofilms dominated by *F. nucleatum* can cause acetylated-polyamine accumulation through dysregulated host polyamine metabolism. These immunosuppressive metabolites inhibit M1 macrophage polarization, promote MDSC recruitment, and weaken T cell infiltration, thereby creating an immunosuppressive TME and driving treatment resistance (61). ETBF upregulates spermidine oxidase (SMO) expression in colonic epithelial cells through secreted *Bacteroides fragilis* toxin (BFT). SMO is a key enzyme in polyamine catabolism, and its upregulation increases ROS generation and DNA damage; use of the polyamine-catabolism inhibitor MDL72527 can markedly reduce ETBF-induced chronic inflammation and malignant proliferation (62). In addition, colibactin produced by *E. coli* induces DNA double-strand breaks and genomic instability (63). Colibactin tolerance has also been associated with irinotecan resistance in CRC cells (45). In contrast to the above pro-resistance microbiota, small RNAs in extracellular vesicles derived from *Lactobacillus murinus* can downregulate host polyamine metabolism by targeting the expression of polyamine-metabolism enzymes. In CRC mouse models and human CRC samples, the abundance of these small RNAs is decreased and is accompanied by increased polyamine levels (64).

Dysregulated tryptophan metabolism has been widely reported in CRC, but it comprises biologically distinct microbial and host-cell pathways. In the microbial branch, gut bacteria convert unabsorbed tryptophan into indole derivatives that can regulate epithelial and tumor signaling (65). Trans-3-indoleacrylic acid (IDA) produced by *Peptostreptococcus anaerobius* acts as an endogenous aryl hydrocarbon receptor (AHR) ligand and transcriptionally upregulates ALDH1A3. ALDH1A3 uses retinal as a substrate to generate NADH, which supports FSP1-mediated reduction of coenzyme Q_10_, suppresses ferroptosis, and promotes CRC progression (66). By contrast, the indoleamine 2,3-dioxygenase 1 (IDO1)–kynurenine pathway described in CRC is mediated primarily by host cells rather than representing a microbiota-specific metabolic pathway. IDO1, expressed by neoplastic epithelial and infiltrating myeloid cells, initiates the conversion of tryptophan through N-formylkynurenine to kynurenine. In preclinical CRC models, kynurenine produced by IDO1-expressing CRC cells downregulates IGFBP5/p53 signaling and attenuates 5-FU-induced cellular senescence, accompanied by reduced p16, p21, p53, and cyclin D1 expression, thereby conferring resistance to 5-FU (67). Host IDO1-derived kynurenine-pathway metabolites also activate PI3K–AKT signaling in neoplastic colon epithelium, promoting proliferation and resistance to apoptosis (68). Microbial metabolites can nevertheless regulate this host pathway. Fong et al. demonstrated that indole-3-carboxylic acid (ICA) produced by *Lactobacillus gallinarum* reduced tumor IDO1 expression and kynurenine production. ICA also competed with kynurenine for AHR binding in CD4^+^ T cells, inhibited Treg differentiation, and enhanced intratumoral CD8^+^ T cell effector function (69). Although these effects were demonstrated in anti-PD-1 treatment models, they establish that microbiota-derived metabolites can restrain the host IDO1/kynurenine/AHR axis and provide a rationale for investigating microbiota-mediated IDO1 suppression as a strategy to counter IDO1-dependent chemoresistance in CRC (**Figure 2**).

**Figure 2:**
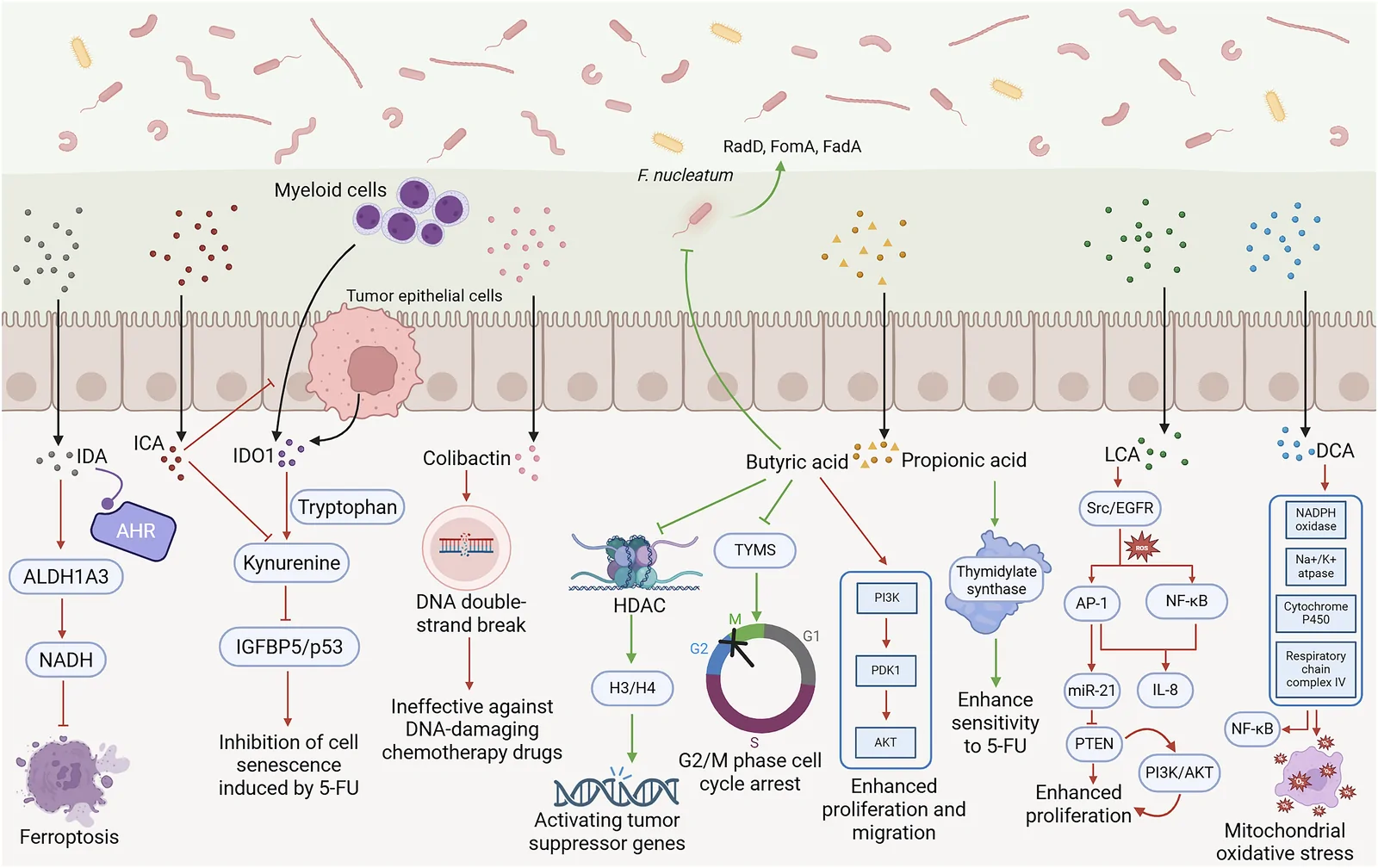
Microbial and host cell metabolite pathways modulate chemotherapy response in colorectal cancer. Microbiota-derived and host cell tryptophan metabolism are shown as distinct but interacting pathways. IDA derived from Peptostreptococcus anaerobius activates AHR–ALDH1A3 signaling and supports FSP1-dependent suppression of ferroptosis, whereas host cell IDO1 generates kynurenine, which can attenuate 5-FU-induced senescence. Microbiota-derived ICA links these pathways by suppressing tumor IDO1 expression and kynurenine production. Other metabolites shown include colibactin, butyrate, propionate, lithocholic acid, and deoxycholic acid, which influence DNA damage, epigenetic regulation, oxidative stress, ferroptosis, proliferative signaling, and chemotherapy sensitivity. Microbiota-derived metabolites are positioned in the gut-lumen compartment, whereas host enzymes and host-derived metabolites are positioned within epithelial, tumor, or immune cell compartments. The figure primarily integrates mechanistic evidence from cellular and animal models. Experimental metabolite administration establishes compound-level biological activity and defines mechanistic pathways that can be evaluated in relation to endogenous microbiota-dependent exposure. Physiological relevance depends on whether comparable concentrations are achieved in vivo. Arrowheads indicate promotion or activation and blunt-ended lines indicate inhibition. Red lines indicate resistance-promoting or sensitivity-reducing effects, whereas green lines indicate resistance-inhibiting or sensitivity-enhancing effects. 5-FU, 5-fluorouracil; AHR, aryl hydrocarbon receptor; AKT, protein kinase B; ALDH1A3, aldehyde dehydrogenase 1 family member A3; AP-1, activator protein 1; CoQ_10_, coenzyme Q_10_; DCA, deoxycholic acid; EGFR, epidermal growth factor receptor; FSP1, ferroptosis suppressor protein 1; HDAC, histone deacetylase; ICA, indole-3-carboxylic acid; IDA, trans-3-indoleacrylic acid; IDO1, indoleamine 2,3-dioxygenase 1; IL-8, interleukin 8; Kyn, kynurenine; LCA, lithocholic acid; NF-κB, nuclear factor kappa B; PI3K, phosphoinositide 3-kinase; ROS, reactive oxygen species; Treg, regulatory T cell; TYMS, thymidylate synthase.

### 2.3 Drug-induced gut microbiome remodeling and its feedback effects on CRC chemotherapy

Remodeling of the gut microbiome by chemotherapeutic drugs is not a simple change in microbial abundance but a multilayered reorganization involving microbial composition, metabolic function, and immune regulation. Its feedback effects can further affect drug efficacy and toxicity, forming a complex regulatory loop.

At clinically equivalent doses, 5-FU, irinotecan, and oxaliplatin can all cause intestinal injury and microbiota dysbiosis, but the patterns of drug-induced microbial changes differ; oxaliplatin causes the most severe intestinal injury and dysbiosis (70). PICRUSt functional-prediction analysis showed that dysbiosis induced by 5-FU, irinotecan, and oxaliplatin is associated with NOD-like signaling pathways. Protein-expression analysis confirmed that oxaliplatin most strongly activates the NOD/RIP2/NF-κB signaling pathway, and 13 bacterial genera, including *Bifidobacterium*, *Akkermansia*, and *Escherichia/Shigella*, are highly correlated with activation of this pathway and with different degrees of intestinal injury (70). The gut microbiota of patients with CRC is also significantly remodeled after CAPOX/mFOLFOX6 adjuvant chemotherapy, with increased relative abundance of proinflammatory genera such as *Porphyromonas*, *Peptostreptococcus*, and *Parvimonas* and decreased abundance of beneficial genera such as *Faecalibacterium* and *Ruminococcaceae*. At the same time, serum anti-lipopolysaccharide (LPS) IgA/IgG levels decrease significantly, suggesting reduced bacterial translocation, whereas expression of tumor-suppressive miR-143 and miR-145 increases more than twofold and is positively correlated with microbial changes, indicating that while eliminating tumors, chemotherapy also exerts dual therapeutic effects by remodeling the microbiota and regulating host miRNAs (71). In addition, dietary factors during chemotherapy can regulate the direction of microbial remodeling. A high-salt diet markedly reduces the anti-CRC efficacy of FOLFOX in mice through gut-microbiota tryptophan metabolism (72); a specific tryptophan metabolite (SK) inhibits FOLFOX efficacy, whereas IPA enhances efficacy through macrophages (72).

Chemotherapy-induced microbial remodeling is not a unidirectional process. The remodeled microbiota can feed back on chemotherapy through microbial drug biotransformation, metabolite signaling, and immune regulation, thereby altering effective drug activity, tumor-cell sensitivity, or treatment toxicity. At the level of drug metabolism, the preTA operon is significantly enriched in gut microbiota during fluoropyrimidine treatment, and preTA-expressing bacteria can convert active 5-FU to inactive metabolites, reducing the effective drug concentration locally in the intestine. Germ-free and antibiotic-treated mice show greater fluoropyrimidine toxicity, whereas transplantation of preTA^+^ *Escherichia coli* or feces from patients with CRC with high preTA expression can rescue this toxicity (21) (**Table 1**). During irinotecan treatment, β-glucuronidase (GUS) derived from the gut microbiota can reactivate SN-38G, which has been detoxified by the liver and excreted into the intestine, to the toxic form SN-38, aggravating delayed diarrhea, a dose-limiting toxicity (73) (**Figure 3B**; **Table 1**). *Bacteroides intestinalis* influences PI3K/AKT-dependent proliferation of intestinal stem cells through the tryptophan metabolite IAA and sensitizes intestinal epithelium to chemical injury caused by the active irinotecan metabolite SN-38 (75). In patients with severe oxaliplatin-induced intestinal toxicity, serum L-kynurenine levels are increased. L-kynurenine accumulation causes dysbiosis characterized by depletion of *Lactobacillus johnsonii*, subsequently activates the TNF-α/JNK signaling pathway, and ultimately causes intestinal epithelial-cell apoptosis; pharmacologic inhibition or engineered reduction of L-kynurenine can alleviate chemotherapy-induced intestinal injury (76). Together, these findings reveal a feedback loop in which chemotherapeutic drugs select specific microbial communities and metabolic functions, microbial enzymes or metabolites alter drug activity, tumor-cell response, or treatment toxicity, and subsequent treatment cycles further remodel the microbiota. This feedback loop provides a rationale for evaluating microbiota monitoring and mechanism-matched interventions during chemotherapy (**Figure 3**; **Table 1**).

**Figure 3:**
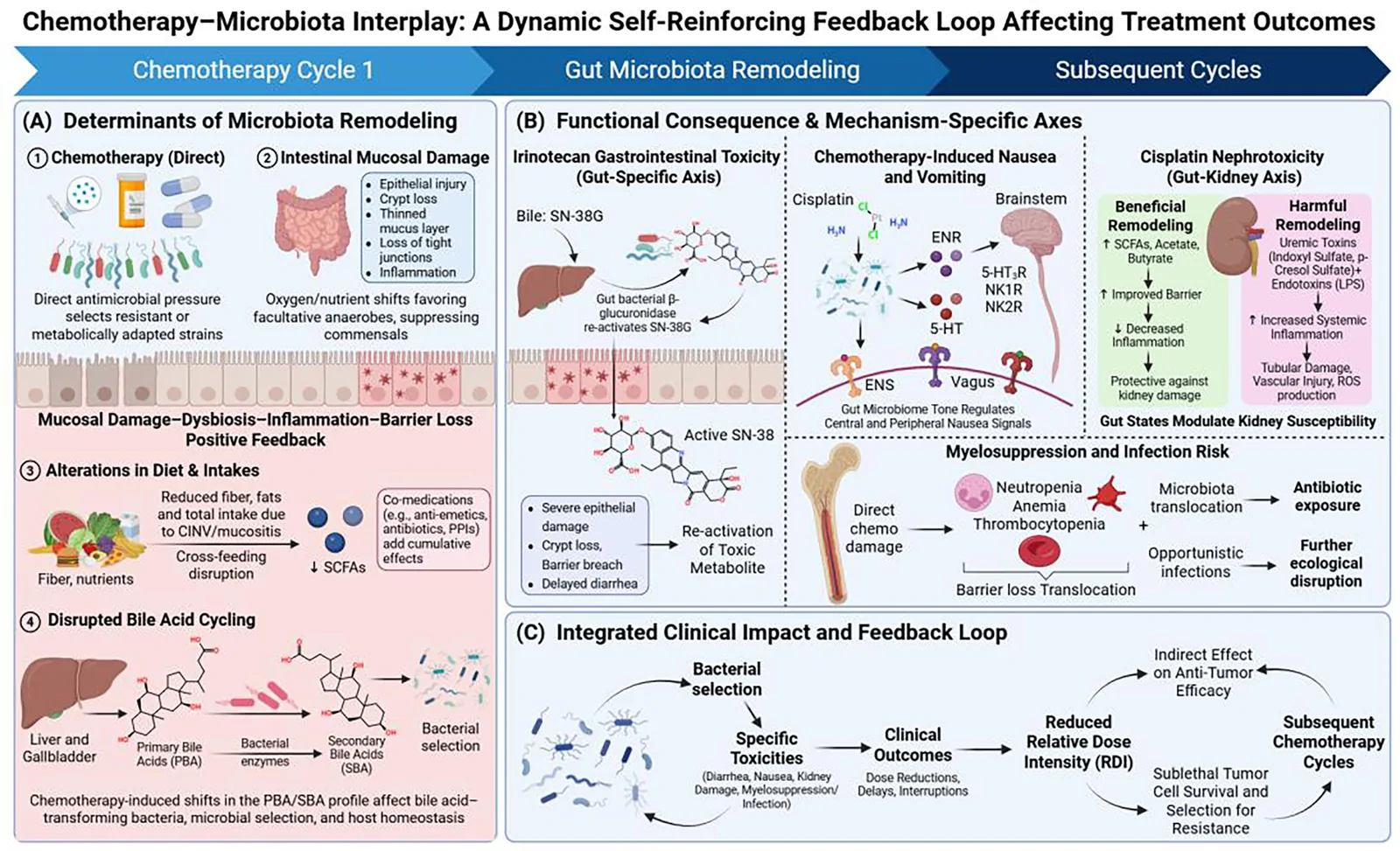
Chemotherapy–microbiota interplay forms a dynamic self-reinforcing feedback loop that influences treatment outcomes. **(A)** Chemotherapy directly selects resistant microorganisms and indirectly reshapes the gut microbiota through mucosal injury, reduced dietary intake, concomitant medications, and disruption of bile-acid cycling, promoting dysbiosis, inflammation, and barrier dysfunction. **(B)** The remodeled microbiota alters treatment toxicity through mechanism-specific pathways, including reactivation of SN-38 by bacterial β-glucuronidase, gut–brain regulation of chemotherapy-induced nausea and vomiting, metabolite-dependent modulation of cisplatin nephrotoxicity (74), and microbial translocation following myelosuppression. **(C)** These toxicities can cause dose reduction, treatment delay, or interruption, thereby lowering relative dose intensity and indirectly compromising tumor control. Subsequent chemotherapy cycles can further remodel the microbiota and reinforce this feedback loop. The figure integrates evidence from mechanistic preclinical studies and clinical toxicity associations; toxicity-mediated reductions in treatment exposure are distinguished from direct tumor-cell chemoresistance. CINV, chemotherapy-induced nausea and vomiting; GUS, β-glucuronidase; RDI, relative dose intensity; SCFAs, short-chain fatty acids; SN-38, 7-ethyl-10-hydroxycamptothecin; SN-38G, SN-38 glucuronide. .

## 3 Regulatory roles of the gut microbiome in lung cancer chemotherapy response

Although targeted therapies and immunotherapies have substantially changed treatment pathways for some patients with lung cancer, platinum-based combination chemotherapy remains an important therapeutic backbone for advanced non-small cell lung cancer (NSCLC) and small cell lung cancer and is widely used in chemoimmunotherapy regimens (77). Clinically, the same cisplatin- or carboplatin-containing regimen can produce markedly different outcomes, including durable remission, transient stability, or primary progression (78). Available evidence suggests that pretreatment microbial composition and metabolic function may preset the host immune and metabolic state during chemotherapy, whereas chemotherapy-induced mucosal injury can in turn remodel the microbiota and further affect control of residual lesions, treatment toxicity, and treatment completion (4) (**Figure 4**; **Table 2**).

**Figure 4:**
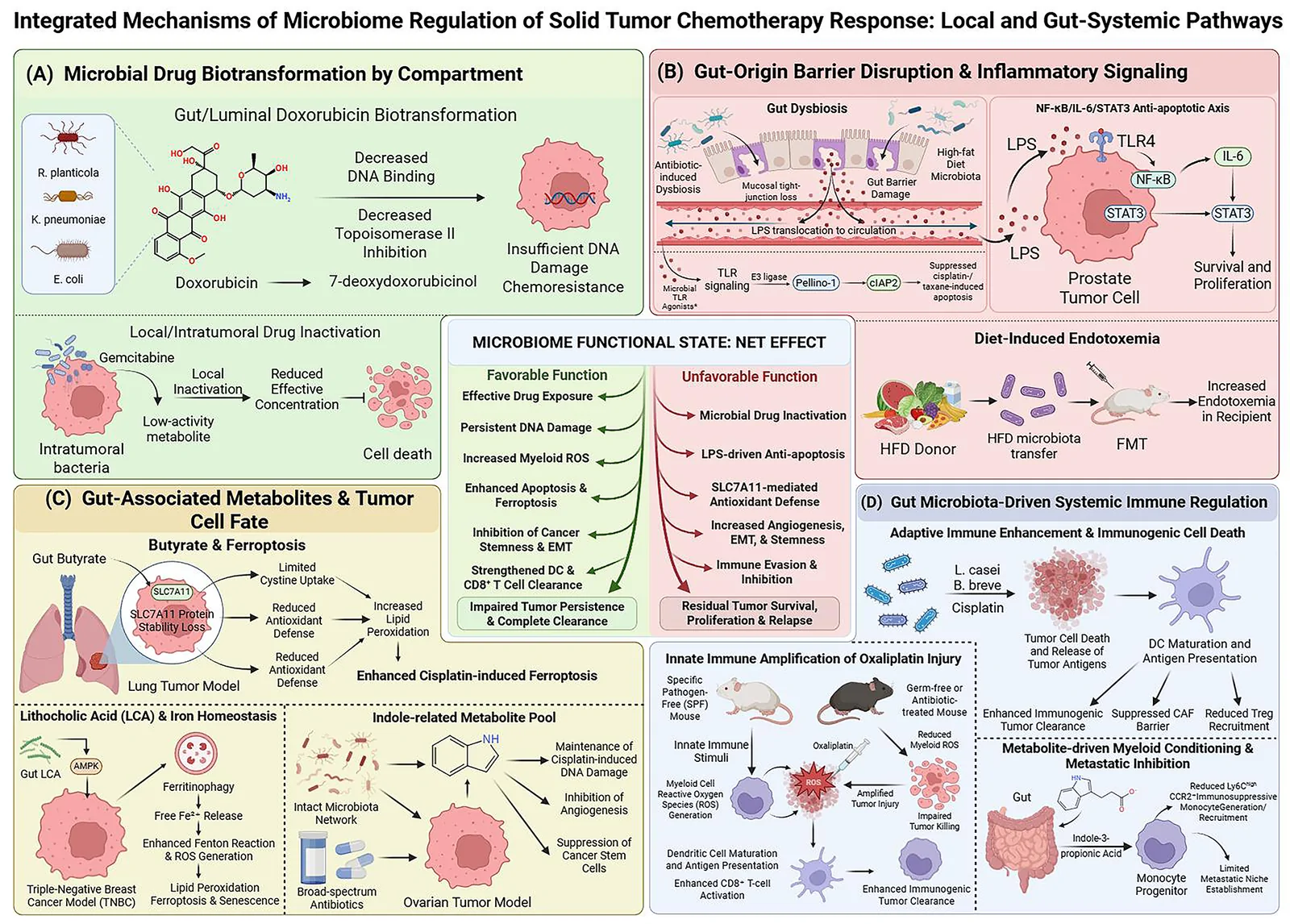
Integrated local and gut-derived systemic mechanisms by which the microbiome regulates chemotherapy responses in solid tumors. **(A)** Microbial drug biotransformation is compartment dependent. Gut/luminal bacteria can metabolize doxorubicin into less active derivatives, whereas intratumoral bacteria can locally inactivate gemcitabine and reduce effective drug exposure. **(B)** Gut dysbiosis and barrier disruption promote the translocation of microbial products such as lipopolysaccharide (LPS) into the circulation, activating tumor-cell inflammatory and anti-apoptotic signaling through the NF-κB/IL-6/STAT3 axis; microbiota associated with a high-fat diet can further promote systemic endotoxemia. Microbial TLR agonists* can also activate the Pellino-1–cIAP2 anti-apoptotic pathway. **(C)** Gut-associated metabolites can regulate tumor-cell fate. Butyrate and lithocholic acid illustrate direct metabolite-responsive mechanisms identified primarily through experimental compound administration, whereas indole-related pathways also include microbiota-perturbation and metabolite-rescue evidence. Experimental metabolite treatment establishes biological sufficiency, while the physiological relevance of these pathways depends on whether endogenous microbiota-dependent exposure reaches comparable concentrations *in vivo*. **(D)** Gut microbiota regulate systemic antitumor immunity through dendritic cell maturation, CD8^+^ T cell activation, microbiota-dependent myeloid reactive oxygen species generation, and metabolite-driven myeloid remodeling, thereby influencing tumor clearance and metastatic progression. The central panel summarizes the net balance between favorable and unfavorable microbiome-associated functions. Local/intratumoral mechanisms arise within tumor tissue or another organ-specific niche, whereas gut-derived systemic mechanisms originate in the intestinal microbiota and act through circulating microbial products, metabolites, or immune pathways. The figure integrates evidence from in vitro mechanistic studies, animal models, microbiota-transfer or metabolite-perturbation experiments, and selected clinical associations. Cross-cancer mechanisms provide transferable mechanistic frameworks for interpreting chemotherapy–microbiome interactions, while cancer-specific validation determines their applicability to individual tumor types. *The specific microbial compartment of origin for TLR/Pellino-1/cIAP2 signaling has not been established. AMPK, AMP-activated protein kinase; cIAP2, cellular inhibitor of apoptosis protein 2; DC, dendritic cell; EMT, epithelial–mesenchymal transition; FMT, fecal microbiota transplantation; HFD, high-fat diet; LCA, lithocholic acid; LPS, lipopolysaccharide; NF-κB, nuclear factor kappa B; ROS, reactive oxygen species; SLC7A11, solute carrier family 7 member 11; STAT3, signal transducer and activator of transcription 3; TLR, Toll-like receptor.

**Table 2:** Microbiota-associated mechanisms influencing chemotherapy response in non-colorectal cancers.

Cancer type	Microbial factor and compartment	Experimental model and chemotherapy	Core mechanism	Chemotherapy-related finding	Evidence type	Reference
Lung cancer	Gut microbiota: *Akkermansia muciniphila*	Lewis lung carcinoma mice; oral *A. muciniphila* plus cisplatin	Reduced peripheral/splenic CD4^+^CD25^+^Foxp3^+^ Treg cells and modulated Fas-related death signaling.	May enhance cisplatin response; combination further reduced tumor volume versus cisplatin alone.	Animal study	(79)
Lung cancer	TLR/Pellino-1/cIAP2 signaling	A549 and H1299 cells; cisplatin and paclitaxel	TLR signaling induces Pellino-1, which stabilizes cIAP2 through Lys63-linked polyubiquitination and limits chemotherapy-induced caspase cleavage.	Raised apoptotic threshold; Pellino-1 or cIAP2 knockdown restored drug sensitivity.	*In vitro* mechanistic study; microbial source not established	(80)
Lung cancer	Butyrate (microbial SCFA; exogenous metabolite treatment)	A549/H1299 cells and xenografts; butyrate plus cisplatin	Promotes SLC7A11 ubiquitination/degradation, Fe^2+^-associated lipid peroxidation and ferroptosis; suppresses stemness and EMT.	Enhanced cisplatin growth inhibition relative to cisplatin alone.	*In vitro* and xenograft metabolite-treatment study	(81)
Triple-negative breast cancer	Gut microbiota from donors fed a high-fat diet and circulating LPS	Orthotopic 4T1 model; FMT from donors fed a high-fat diet or LPS plus doxorubicin	Microbiota-associated circulating LPS reduced doxorubicin-induced cleaved caspase-3 and tumor-cell apoptosis.	Transferred a low-response phenotype and promoted tumor growth and lung metastasis.	FMT, metabolite add-back and animal studies	(82)
Breast cancer and pan-tumor models	Small-intestinal Gram-positive commensals	Germ-free, antibiotic-treated and conventional mice; cyclophosphamide	Cyclophosphamide increases small-intestinal permeability, enabling commensal translocation to secondary lymphoid organs and pathogenic Th17/memory Th1 responses.	Microbiota-dependent immune potentiation: germ-free or antibiotic treatment weakened antitumor activity; Th17 transfer partly restored efficacy.	Animal immunologic mechanism study	(12)
Breast cancer-related chemotherapy	Gut bacteria: *Raoultella planticola*, *Klebsiella pneumoniae* and *Escherichia coli*	Anaerobic culture and drug biotransformation assays; doxorubicin	Reductive deglycosylation or molybdopterin-dependent enzyme systems convert doxorubicin to low-activity products, including 7-deoxydoxorubicinol and 7-deoxydoxorubicinolone.	Direct microbial reduction of doxorubicin activity *in vitro*, supporting a potential microbiota-dependent drug-biotransformation mechanism.	*In vitro* drug biotransformation study	(83)
Ovarian cancer	Antibiotic-disrupted gut microbiota and indole-related metabolites	ID8, ID8-VEGF and OV81 models; cisplatin	Microbiota disruption reduced cisplatin-induced DNA damage, increased angiogenesis and post-treatment cancer stem cells, with lower indole-3-propionic acid, indoxyl sulfate and hippurate.	Weakened cisplatin efficacy; intact-donor FMT partly restored sensitivity and survival.	Animal models and FMT rescue	(84)
Cervical cancer	Local cervical/tumor-associated microbiota: *L. iners* and L-lactate	Patient-derived organoids and primary cervical cancer cells; radiation, gemcitabine, cisplatin and 5-FU	L-lactate remodels GLUT1, MCT1/MCT4, lactate use and redox networks, increasing metabolic and redox adaptation.	Increased tolerance to radiation and gemcitabine; effects on cisplatin and 5-FU were cell-context dependent.	Patient samples, organoids and cell mechanistic study	(85)
Prostate cancer	Gut dysbiosis: Proteobacteria expansion, barrier leakage, and circulating/tumoral LPS	RM-1 subcutaneous/orthotopic models and DU145 cells; docetaxel	Dysbiosis increases permeability and tumoral LPS, activating NF-κB/IL-6/STAT3 and upregulating Bcl-2 and Survivin to suppress apoptosis.	Weakened docetaxel activity; FMT transferred low response and STAT3 inhibition partly restored efficacy.	Patient association, FMT, animal and cell pathway studies	(86)
Pancreatic ductal adenocarcinoma	Intratumoral Gammaproteobacteria with long isoform cytidine deaminase	Tumor isolates, cells and mouse models; gemcitabine	Bacterial cytidine deaminase converts gemcitabine to inactive 2’,2’-difluorodeoxyuridine.	Directly inactivated local gemcitabine and conferred drug tolerance; antibacterial treatment restored activity in mice. Provides a plausible local drug-inactivation mechanism with potential relevance to urothelial carcinoma; cancer-specific applicability remains to be defined.	Tumor isolates, *in vitro* and animal studies	(9)

5-FU, 5-fluorouracil; Bcl-2, B-cell lymphoma 2; CD4, cluster of differentiation 4; CD25, cluster of differentiation 25; cIAP2, cellular inhibitor of apoptosis protein 2; EMT, epithelial–mesenchymal transition; Fas, Fas cell surface death receptor; FMT, fecal microbiota transplantation; Foxp3, forkhead box P3; GLUT1, glucose transporter 1; IL-6, interleukin 6; LPS, lipopolysaccharide; MCT1, monocarboxylate transporter 1; MCT4, monocarboxylate transporter 4; NF-κB, nuclear factor kappa B; SLC7A11, solute carrier family 7 member 11; STAT3, signal transducer and activator of transcription 3; Th1, T helper 1 cell; Th17, T helper 17 cell; TLR, Toll-like receptor; TNBC, triple-negative breast cancer; Treg, regulatory T cell; VEGF, vascular endothelial growth factor.

### 3.1 Pretreatment microbial characteristics, dynamic changes during treatment, and lung cancer chemotherapy response

Lung-cancer cohorts indicate that microecologic information associated with chemotherapy response may lie mainly in low-abundance species and specific metabolic functions, rather than in unidirectional changes in overall microbial diversity. Zhao et al. performed baseline fecal metagenomic sequencing in 64 treatment-naive patients with locally advanced or advanced lung cancer. Patients received first-line pemetrexed, paclitaxel, gemcitabine, or etoposide combined with cisplatin or carboplatin and were classified according to Response Evaluation Criteria in Solid Tumors (RECIST) version 1.1 as 33 responders and 31 nonresponders (87). The Shannon index and Bray–Curtis β-diversity did not differ significantly between groups, but species-level and functional characteristics differed: *Streptococcus mutans*, *Enterococcus casseliflavus*, *Acidobacteria*, and *Granulicella* were relatively enriched in responders, whereas *Leuconostoc lactis*, *Eubacterium siraeum*, and *Rothia dentocariosa* were more abundant in nonresponders. Functional analysis showed enrichment of the L-glutamate-degradation-VIII pathway in responders, whereas the reductive tricarboxylic-acid cycle and hexitol-fermentation pathways producing lactate, formate, ethanol, and acetate were enriched in nonresponders. Median progression-free survival in this cohort was 7 months, and patients who had used antibiotics, probiotics, or glucocorticoids within 6 months before sampling were excluded. These findings suggest that microecologic phenotypes associated with lung-cancer chemotherapy response may be better characterized by combinations of specific species and functional pathways than by overall diversity metrics alone (87).

Longitudinal sampling during treatment further indicates that, in addition to baseline microbial characteristics, the stability of community composition and functional drift during chemotherapy are also associated with treatment outcomes. A prospective study of 45 patients with advanced NSCLC collected fecal samples before treatment and after completion of two cycles of platinum-doublet chemotherapy (88). Several *Bacteroides* species were relatively enriched at baseline in responders, whereas six major metabolic pathways were relatively enhanced in nonresponders. After chemotherapy, the abundances of *Streptococcaceae*, *Lactobacillaceae*, and *Leuconostocaceae* increased, and *Leuconostocaceae* was also associated with gastrointestinal adverse events during treatment (88). These findings indicate that chemotherapy was accompanied by changes in specific microbial taxa and metabolic functions associated with efficacy and toxicity.

A Thai cohort reported in 2026 included 27 patients with advanced NSCLC, of whom 26 were evaluable for efficacy; 13 patients each were assigned to the disease-progression and clinical-benefit groups (89). Baseline α-diversity and β-diversity did not differ significantly between groups, and *Akkermansia muciniphila* showed only a nonsignificant enrichment trend in the clinical-benefit group. Among 20 patients with paired samples, ACE and Chao1 indices declined after chemotherapy, whereas the Shannon and Simpson indices and overall β-diversity did not change significantly (89). Taken together, these cohorts show that the direction of individual differential taxa is not fully consistent across studies, possibly because of differences in population region, dietary patterns, sequencing resolution, chemotherapy combinations, and sampling time points. A relatively stable conclusion is that microecologic phenotypes during platinum treatment of lung cancer are more likely to comprise specific strains, metabolic functions, and community stability during treatment, rather than simply being summarized as “the higher the microbial diversity, the better the chemotherapy efficacy (**Table 3A**).

**Table 3:** Microbiota-associated and metabolic biomarkers of chemotherapy efficacy and toxicity in non-colorectal cancers.

A. Biomarkers of chemotherapy efficacy and outcome.						
Cancer type	Biomarker type and feature	Study population and sampling time	Treatment and endpoint	Key association or model performance	Potential clinical role	Reference
Lung cancer	Pretreatment microbial signature: multispecies and microbial-function combination	64 treatment-naive locally advanced/advanced patients; pretreatment stool metagenomics	Pemetrexed, paclitaxel, gemcitabine or etoposide plus cisplatin/carboplatin; RECIST 1.1 response	No significant Shannon or Bray-Curtis difference, but species/function profiles differed: responders enriched for *Streptococcus mutans* and *Enterococcus casseliflavus*; nonresponders for *Leuconostoc lactis*, *Eubacterium siraeum* and *Rothia dentocariosa*.	Candidate pretreatment response stratifier; species/function information may exceed overall diversity.	(87)
Non-small cell lung cancer	Pretreatment circulating metabolite panel: kynurenine, tryptophan, and xanthurenic acid	323 participants across discovery and targeted-quantitation cohorts; pretreatment blood	First-line pemetrexed plus cisplatin/carboplatin; sensitive versus resistant	Resistance associated with higher kynurenine and lower tryptophan/xanthurenic acid; random-forest AUC 0.954.	Candidate pretreatment metabolic response classifier; microbial contribution remains unresolved.	(90)
Non-small cell lung cancer	Early dynamic microbial signature: 2-week diversity and *Lachnospiraceae* NK4A136, UCG-003, *Dorea*, *Eubacterium hallii* group, *E. siraeum* group and Muribaculaceae	41 concurrent chemoradiotherapy patients; baseline, week 2 and end of treatment (102 stool samples)	Concurrent chemoradiotherapy; PFS dichotomized at 11 months	Baseline did not stably distinguish outcome; week-2 random-forest AUC 0.87. Phylogenetic diversity and *Dorea* abundance were independently associated with PFS.	Candidate early dynamic marker for overall chemoradiotherapy outcome.	(91)
Triple-negative breast cancer	Pretreatment microbial signature: baseline Shannon diversity	25 non-metastatic patients; stool before neoadjuvant therapy	Weekly paclitaxel for 12 weeks, then epirubicin/cyclophosphamide; most also received carboplatin; pCR	Baseline Shannon diversity was higher in patients achieving pCR (14 pCR vs 11 residual disease; P = 0.049).	Candidate baseline marker for neoadjuvant chemotherapy response.	(92)
Early breast cancer	Pretreatment microbial signature: specific taxonomic features	Prospective cohort across eight institutions; 183 patients	Neoadjuvant systemic therapy; pCR	Overall pCR rate 36.1%; Victivallales and Anaerolineales associated with pCR, whereas Gemellales associated with residual disease; α-diversity did not differ.	Candidate pCR-associated taxonomic signature.	(93)
Breast cancer	Pretreatment microbial signature: species richness, SCFA-related taxa and metabolites	39 chemotherapy-naive patients; baseline shotgun metagenomics and targeted metabolomics	Neoadjuvant chemotherapy; pCR	pCR group (17 vs 22 non-pCR) had greater species richness and enrichment of *Dialister*, *Kineothrix* and *Jutongia*; non-pCR group enriched for *Rothia*, *Leuconostoc* and *Klebsiella*. Acetate, propionate and butyrate correlated with microbial glycolytic pathways.	Candidate multi-omics pCR stratifier.	(94)
Ovarian cancer	Pretreatment microbial signature: diversity plus Angelakisella, *Arenimonas and Roseburia* composition	174 patients; pretreatment 16S rRNA sequencing	Post-debulking carboplatin/paclitaxel; recurrence within 6 months defined platinum resistance	Platinum-resistant group had higher Shannon/Simpson diversity and Proteobacteria; platinum-sensitive group was Firmicutes-dominant; random-forest AUC 0.909.	Candidate pretreatment platinum-sensitivity classifier.	(95)
Epithelial ovarian cancer	Longitudinal ecological stability: on-treatment α-diversity, individual microbial fingerprint and lactate-associated taxa	24 patients; 406 longitudinal samples during neoadjuvant or adjuvant chemotherapy	Platinum chemotherapy; platinum resistance versus sensitivity	Baseline single samples did not stably discriminate outcome. Resistant patients showed progressive diversity loss, fingerprint loss and greater volatility with lactate-producing taxa; sensitive patients were more stable and enriched for Veillonellaceae.	Candidate on-treatment ecological stability marker for platinum response monitoring.	(96)
Locally advanced cervical cancer	Pretreatment microbial signature: baseline Shannon diversity and taxa	55 patients; stool before concurrent chemoradiotherapy	External beam radiation, weekly cisplatin and brachytherapy; recurrence-free and overall survival	Higher baseline Shannon diversity independently associated with longer recurrence-free and overall survival and with increased on-treatment CD4^+^ T cell activation/proliferation.	Candidate baseline marker for cisplatin-based chemoradiotherapy outcome.	(97)
Cervical cancer	Microbiota-immune composite signature: *Escherichia*/*Ruminococcus* ratio plus PD-1^+^CD56^bright^ NK cell frequency	77 women: untreated patients, patients receiving radiotherapy plus platinum chemotherapy, and healthy controls	Radiotherapy plus platinum chemotherapy; survival	Post-treatment dysbiosis and NK cell exhaustion scores were each associated with poorer survival.	Exploratory microbiota-immune composite outcome marker.	(98)
Muscle-invasive bladder cancer	Pretreatment microbial signature: Oscillibacter, *Fusicatenibacter and Bacteroides*-based feature	Multicenter prospective study; 57 patients with complete response data	Cisplatin-based neoadjuvant chemotherapy; pathologic complete response	Microbial feature model predicted complete response with AUC 0.88 (sensitivity 0.70; specificity 0.80), versus clinical-model AUC 0.50.	Candidate neoadjuvant chemotherapy pCR classifier.	(13)

B. Biomarkers of chemotherapy toxicity.						
Cancer type	Biomarker type and microbial feature	Study population and sampling time	Treatment and endpoint	Key association or model performance	Potential clinical role	Reference
Breast cancer	Pretreatment microbial signature: α-diversity and Synergistota/Synergistia/Synergistales taxa	301 patients receiving first-line chemotherapy; pretreatment shotgun metagenomics	First-line chemotherapy; grade ≥3 hematologic toxicity and severe neutropenia	Higher baseline α-diversity was associated with lower risks of severe hematologic toxicity and severe neutropenia (multivariable OR 0.94 for each).	Candidate baseline hematologic-toxicity risk marker.	(99)
Breast cancer	Pretreatment microbial signature: Chao1, β-diversity, Lachnospiraceae/Oscillospiraceae/Ruminococcaceae members and functional pathways	Same 301-patient first-line chemotherapy cohort; pretreatment sampling	First-line chemotherapy; severe nausea and vomiting	Severe nausea/vomiting associated with higher Chao1, specific β-diversity structure, related taxa and the reductive TCA-cycle pathway; *Odoribacter laneus* and L-proline biosynthesis II were linked to lower risk.	Candidate baseline gastrointestinal-toxicity risk feature.	(99)
Breast cancer	Longitudinal ecological stability: baseline α-diversity, *Faecalibacterium* and on-treatment stability	59 patients; serial stool, blood and symptom assessments before, during and after chemotherapy	Chemotherapy; nausea, vomiting and diarrhea	Lower baseline α-diversity and reduced *Faecalibacterium* associated with more severe subsequent gastrointestinal symptoms; Bray-Curtis displacement correlated with diarrhea severity.	Candidate baseline ecological-vulnerability and dynamic gastrointestinal-toxicity marker.	(100)
Early breast cancer	Treatment-emergent multimodal signature: microbiota displacement, SCFAs, bile acids and circulating neurofilament light	70 patients receiving paclitaxel or docetaxel; stool and blood before first and before final infusion	Paclitaxel or docetaxel; peripheral neuropathy	Community displacement correlated with sensory neuropathy severity; neuropathy coincided with lower *Collinsella*, *Faecalibacterium*, *Blautia*, *Dorea* and *Anaerostipes*, lower acetate/butyrate/isovalerate, higher bile acids and increased neurofilament light.	Candidate on-treatment multimodal neurotoxicity-monitoring signature.	(101)
Ovarian cancer	Pretreatment microbial signature: *Bacteroides* caccae, Corynebacteriales, *Corynebacterium and* metabolic pathways	First platinum-taxane treatment; 25 CINV and 25 non-CINV patients; pretreatment sampling	Carboplatin/cisplatin plus paclitaxel; chemotherapy-induced nausea and vomiting (CINV)	CINV group showed enrichment of specific bacteria and abnormalities in pentose phosphate, glutathione and lipoic-acid metabolism; non-CINV FMT attenuated cisplatin-induced pica in rats.	Candidate platinum-associated CINV risk marker; supports involvement of the gut–brain axis.	(102)
Locally advanced cervical cancer	Early dynamic microbial signature: on-treatment gut microbial diversity	35 patients; baseline and weeks 1, 3 and 5	Cisplatin concurrent chemoradiotherapy; EPIC gastrointestinal-function score	Diversity declined during treatment alongside worsening gastrointestinal function; higher diversity at each time point associated with milder symptoms.	Candidate on-treatment gut-tolerance monitoring marker.	(103)

16S rRNA, 16S ribosomal RNA; AUC, area under the receiver operating characteristic curve; CD4, cluster of differentiation 4; CD56, cluster of differentiation 56; CINV, chemotherapy-induced nausea and vomiting; EPIC, Expanded Prostate Cancer Index Composite; FMT, fecal microbiota transplantation; NK, natural killer; OR, odds ratio; pCR, pathological complete response; PD-1, programmed cell death protein 1; PFS, progression-free survival; RECIST 1.1, Response Evaluation Criteria in Solid Tumors version 1.1; SCFAs, short-chain fatty acids; TCA, tricarboxylic acid.

### 3.2 Regulation of platinum sensitivity by the gut microbiome through immune regulation and tumor cell death pathways

Animal and cellular studies provide complementary mechanistic evidence for microbiota-dependent and microbe-responsive pathways that may influence platinum sensitivity. In a Lewis lung cancer mouse model, 50 C57BL/6 mice were divided into a normal control group, tumor model group, cisplatin group, cisplatin plus antibiotic group, and cisplatin plus *A. muciniphila* group (79). *A. muciniphila* was administered by daily gavage at 10^8^ CFU, cisplatin was administered intraperitoneally at 5 mg/kg every other day, and the intervention continued for 5 weeks. Compared with cisplatin alone, combined *A. muciniphila* further reduced tumor volume and was accompanied by reduced Ki-67, p53, and FasL expression, increased Fas expression, and decreased CD4^+^CD25^+^Foxp3^+^ regulatory T cells in peripheral blood and spleen (79). Although some tumor-volume comparisons showed only a trend toward change, this study still supports the possibility that *A. muciniphila* alters cisplatin response by regulating the Treg proportion and Fas-associated death signaling rather than only through direct inhibition of tumor cells (**Figure 4D**; **Table 2**).

Pan-cancer experiments also provide transferable mechanistic insight into microbiota-dependent regulation of platinum efficacy that may be relevant to lung cancer. Iida et al. found that oxaliplatin-induced reactive oxygen species (ROS) generation by tumor-infiltrating myeloid cells was significantly impaired in germ-free mice or mice treated with broad-spectrum antibiotics, resulting in reduced tumor killing and indicating that commensal signals can support platinum-associated myeloid oxidative responses (11). Although characterized in broader tumor models, this sequence in which gut microbiota regulate reactive oxygen species generation by myeloid cells and thereby tumor killing provides a biologically plausible framework for understanding microbiota-dependent modulation of platinum responses in lung cancer, while validation specifically in lung cancer remains warranted (**Figure 4D**).

Pattern-recognition signaling provides a potential interface between microbial products and intrinsic antiapoptotic pathways in lung cancer cells, although the microbial compartment responsible for activating this pathway has not been established. In A549 and H1299 lung cancer cells, stimulation with Toll-like receptor agonists such as lipopolysaccharide increases expression of the E3 ubiquitin ligase Pellino-1. Pellino-1 stabilizes the apoptosis-inhibitory protein cIAP2 through Lys63-linked polyubiquitination, thereby reducing caspase cleavage induced by cisplatin and paclitaxel. Knockdown of Pellino-1 or cIAP2 restores tumor-cell drug sensitivity, whereas re-expression of cIAP2 reverses the sensitizing effect caused by Pellino-1 knockdown (80). These findings define the TLR/Pellino-1/cIAP2 axis as a microbe-responsive anti-apoptotic pathway capable of reducing cisplatin and paclitaxel sensitivity. Because the physiological microbial source of TLR activation remains unresolved, this pathway is best positioned as a mechanistic interface between microbial pattern recognition and tumor-cell survival signaling (**Figure 4B**; **Table 2**).

### 3.3 Microbe-derived metabolites and chemotherapy-induced distant feedback effects

Butyrate is a canonical short-chain fatty acid produced by microbial fermentation, but the lung-cancer evidence discussed here primarily derives from direct experimental exposure to butyrate rather than from manipulation of gut microbial butyrate production. After A549 and H1299 cells and their cancer stem cell subpopulations were treated with butyrate, CD133 expression, ALDH activity, tumorsphere formation, and EMT were inhibited. Mechanistic studies show that butyrate promotes lysosomal Fe^2+^-associated lipid peroxidation and reduces the stability of the cystine transporter SLC7A11 by enhancing ubiquitination. In cells and xenograft tumors, the combination of butyrate and cisplatin produces stronger growth inhibition than cisplatin alone, and interventions targeting SLC7A11 further support the involvement of ferroptosis in this sensitization process (81). These findings establish an exposure-dependent ferroptosis-sensitizing effect of butyrate mediated by SLC7A11 in lung cancer. Its *in vivo* microbiome relevance will depend on whether gut-derived butyrate achieves systemic and intratumoral exposures within the experimentally active range (**Figure 4C**; **Table 2**).

Conversely, intestinal injury caused by chemotherapy may also induce metabolic feedback with distant antitumor effects. Bersier et al. found that intestinal mucosal injury caused by cisplatin, oxaliplatin, or 5-FU can promote expansion of tryptophan-metabolizing bacteria and increase production of indole-3-propionic acid (IPA). IPA entering the circulation can remodel bone-marrow hematopoiesis, reduce Ly6C^high^ CCR2^+^ immunosuppressive monocytes, and establish a myeloid state unfavorable for metastatic-niche formation (104). Across tumor models, this study identifies a chemotherapy-induced pathway in which gut microbiota-derived IPA remodels myeloid states and post-treatment metastatic ecology, providing a transferable systemic mechanism through which intestinal microbial changes can influence the fate of residual disease after chemotherapy (**Figure 4D**).

## 4 Regulatory roles of the gut microbiome in breast cancer chemotherapy response

Neoadjuvant chemotherapy provides an important clinical window for evaluating drug sensitivity and residual resistance in breast cancer, particularly triple-negative breast cancer (TNBC). Anthracyclines, taxanes, cyclophosphamide, and platinum agents constitute the main neoadjuvant regimens for TNBC. Achievement of pathological complete response (pCR) after treatment is usually associated with a lower risk of recurrence, whereas residual lesions reflect persistent survival of tolerant cell populations under treatment pressure (92, 105).

### 4.1 Baseline gut-microbiota characteristics and response to neoadjuvant chemotherapy for breast cancer

Breast-cancer neoadjuvant-treatment cohorts indicate that baseline microbial diversity and specific taxa are associated with chemotherapy response, although the identified signals are not fully consistent across populations. The MOON study included 25 patients with nonmetastatic TNBC, all of whom received weekly paclitaxel for 12 weeks followed sequentially by epirubicin plus cyclophosphamide; 23 patients also received carboplatin (92). According to ypT0/is, N0 criteria, 14 patients achieved pCR and 11 had residual lesions, while 5 could not complete all planned doses because of treatment toxicity. Baseline Shannon diversity was higher in patients with pCR (P = 0.049), whereas body mass index was the main factor separating β-diversity; stable, consistent community drift during treatment was not observed in early paired samples (92). Setouchi Breast Project-14 prospectively enrolled 183 patients with early breast cancer at 8 institutions; the overall pCR rate was 36.1%. Among 54 patients with TNBC, 16 achieved pCR, yielding a pCR rate of 29.6% (93). α-diversity did not differ significantly between the pCR and non-pCR groups, but *Victivallales* and *Anaerolineales* were associated with pCR (both P = 0.001), whereas *Gemellales* was associated with residual lesions (P = 0.002) (93). These findings indicate that microecologic characteristics of breast-cancer chemotherapy response may be more strongly reflected by specific microbiota and functional combinations than by unidirectional changes in overall diversity.

Integrated species and metabolite analyses further localize response-associated signals to functional groups with short-chain fatty acid and bile acid metabolic capacities. In a shotgun-metagenomic and targeted-metabolomic study of 39 treatment-naive patients with breast cancer, 17 achieved pCR and 22 did not (94). Baseline species richness was higher in the pCR group (P = 0.040), with enrichment of *Dialister*, *Kineothrix*, and *Jutongia*, whereas *Rothia*, *Leuconostoc*, and *Klebsiella* were enriched in the non-pCR group. Acetate, propionate, and butyrate concentrations were positively correlated with microbial glucose-degradation pathways. Deoxycholic acid levels were relatively higher in the non-pCR group, but this difference was no longer statistically significant after multiple-comparison correction; the direction of change in a 15-patient TNBC subgroup was broadly consistent with that in the overall cohort (94). The enrichment of SCFA-associated microbiota and metabolic features in patients with favorable response identifies a metabolic profile associated with chemotherapy outcome; epithelial-barrier maintenance, immune regulation, and suppression of proinflammatory signaling provide plausible biological contexts for this association.

Li et al. performed baseline fecal metagenomic sequencing in 23 patients with invasive breast cancer, of whom 18 had effective treatment and 5 had ineffective treatment. The low-response group showed reduced microbial diversity and abundance, fewer butyrate-producing and bacteria producing indole-3-propionic acid, and increased potential pathogens; a random-forest signature comprising *Coprococcus*, *Dorea*, and uncultured *Ruminococcus* was positively correlated with CD4^+^ T cell numbers in peripheral blood and tumor tissue (106). Reduced representation of butyrate-producing and indole-metabolizing bacteria was associated with the low-response phenotype and with CD4^+^ T cell features, linking these microbial functions to systemic immune status during treatment. This cohort included different molecular subtypes of breast cancer, and some patients also received trastuzumab; its microbiota characteristics therefore reflect the combined effects of cytotoxic treatment, targeted therapy, and host immune background (**Table 3A**).

### 4.2 Chemotherapy sensitivity mediated by the intestinal barrier and immune regulation

Disruption of the intestinal barrier and the resulting endotoxemia can raise the tolerance threshold of TNBC to anthracycline drugs. Bawaneh et al. established orthotopic 4T1 TNBC models in 115 female BALB/c mice and altered the intestinal microecology with broad-spectrum antibiotics, fecal microbiota transplantation from donors fed a high-fat diet, or exogenous lipopolysaccharide (82). Broad-spectrum antibiotic treatment enhanced the tumor-inhibitory effect of doxorubicin, whereas fecal microbiota transplantation from donors fed a high-fat diet increased circulating lipopolysaccharide, reduced doxorubicin-induced cleaved caspase-3 expression, and promoted tumor growth and lung metastasis. Weekly administration of 5 mg/kg exogenous lipopolysaccharide reproduced the low-apoptosis, low-response phenotype, while mice responsive to doxorubicin had higher pretreatment abundance of *Akkermansia muciniphila* (82) (**Figure 4B**; **Table 2**).

The phenotype transfer achieved by fecal microbiota transplantation and lipopolysaccharide reconstitution indicates that microbiota associated with a high-fat diet can reduce doxorubicin efficacy by impairing the intestinal barrier, increasing circulating lipopolysaccharide, and attenuating tumor-cell apoptosis. Circulating lipopolysaccharide may raise the survival threshold of tumor cells through TLR-related inflammatory signaling while promoting immunosuppression and formation of a metastatic ecosystem. Higher pretreatment abundance of *A. muciniphila* was associated with doxorubicin responsiveness in this model, although the mechanisms underlying this association require further clarification (82).

Cyclophosphamide-induced mucosal disturbance can produce an immune-amplifying effect distinct from endotoxemia. Viaud et al. found that cyclophosphamide increases small-intestinal permeability, promotes translocation of some Gram-positive commensals to secondary lymphoid organs, and induces pathogenic Th17 cell and memory Th1 cell responses. Germ-free status or antibiotic treatment weakens the antitumor effect of cyclophosphamide, whereas adoptive transfer of pathogenic Th17 cells can partially restore treatment efficacy (12). The resulting pathway links mucosal injury to commensal translocation and subsequent effector T cell activation, indicating that chemotherapy-induced changes in the intestinal barrier are not merely toxicity events; their biological consequences depend on the composition of translocating microbiota and host immune responses to microbial antigens (**Figure 4D**; **Table 2**).

Across broader tumor models, this sequence of mucosal injury, commensal translocation, and effector T cell activation provides a transferable mechanism for microbiota-dependent amplification of cyclophosphamide activity and a mechanistic framework for evaluating this interaction in breast cancer.

### 4.3 Microbial metabolites and chemotherapeutic-drug biotransformation

Lithocholic acid (LCA) is a secondary bile acid that can be generated through gut microbial metabolism, but the TNBC evidence discussed here primarily reflects direct experimental exposure to LCA. In TNBC cells, LCA activates AMPK signaling, promotes ferritinophagy, increases intracellular free iron and oxidative stress, and thereby induces ferroptosis and cellular senescence (107). Fe^2+^ released by ferritin degradation can promote the Fenton reaction and lipid peroxidation, whereas AMPK activation may also limit anabolic metabolism and energy supply in tumor cells (**Figure 4C**). These experiments establish an LCA-responsive ferroptotic program in TNBC cells. Its microbiome relevance depends on whether physiologically generated gut-derived LCA achieves circulating and intratumoral concentrations within the experimentally active range (**Figure 4C**).

Intestinal bacteria can also reduce the activity of chemotherapeutic drugs through direct biotransformation. Under anaerobic culture conditions, Yan et al. found that *Raoultella planticola* can reductively deglycosylate doxorubicin into 7-deoxydoxorubicinol and 7-deoxydoxorubicinolone; *Klebsiella pneumoniae* and *Escherichia coli* BW25113 can also degrade doxorubicin through a molybdopterin-dependent enzyme system. Compared with the parent drug, these metabolites show markedly lower toxicity to *Caenorhabditis elegans* (83). Disruption of the glycosidic structure of doxorubicin reduces its DNA-binding and inhibitory activity against topoisomerase II, establishing the capacity of intestinal bacteria to generate low-activity doxorubicin metabolites under anaerobic conditions (**Figure 4A**; **Table 2**). Because doxorubicin is administered intravenously, a gut-microbiome effect on systemic drug exposure would most plausibly arise after hepatobiliary delivery of doxorubicin or reabsorbable metabolites into the intestinal lumen. Bacterial deglycosylation during enterohepatic recirculation could reduce reabsorption and shift elimination toward fecal clearance (108), positioning microbial doxorubicin metabolism as a testable pharmacokinetic mechanism linking intestinal bacterial biotransformation with systemic drug disposition. Paired measurement of parent doxorubicin and 7-deoxy metabolites in plasma, bile or feces, and tumor tissue, together with systemic exposure, clearance, and intratumoral drug concentrations, would determine the contribution of this pathway in patients.

### 4.4 Effects of the gut microbiome on chemotherapy toxicity and treatment completion

The gut microbiome can indirectly alter the actual dose intensity of breast-cancer chemotherapy by influencing hematologic toxicity and intestinal homeostasis. Baseline 16S rRNA sequencing in 81 treatment-naive patients with breast cancer showed that severe anemia was associated with enrichment of *Eubacteriaceae*, *Anaerofilum*, and *Eubacterium*, with Cohen’s d values of 1.38, 1.42, and 1.38, respectively; grade 4 neutropenia was associated with depletion of the *Eubacterium_hallii_group* (d = −1.08), and thrombocytopenia was associated with an approximately 53-fold decrease in *Eubacterium limosum* (d = −1.60). After false discovery rate correction, 42 taxa were associated with specific treatment toxicities, with effect sizes ranging from −1.60 to 1.67 (109).

These toxicity-associated microbial features may serve as markers of hematopoietic tolerance, with SCFA production, vitamin and amino-acid metabolism, bone-marrow immune regulation, and intestinal-barrier integrity representing plausible mechanistic links. Severe anemia, neutropenia, and thrombocytopenia can cause treatment delay, dose reduction, or premature discontinuation, thereby reducing relative dose intensity and potentially compromising tumor control. Baseline microbiota may therefore provide information about treatment tolerance and the likelihood of completing adequate-dose chemotherapy (109) (**Figure 3C**; **Table 3B**).

Paclitaxel can also actively remodel the intestinal microbiota and the gut–liver bile-acid circulation. Mouse studies show that paclitaxel treatment is accompanied by altered microbial structure, abnormal bile-acid absorption, and impaired intestinal homeostasis (110). Changes in the bile-acid pool can further affect the intestinal epithelial barrier, hepatic metabolism, and systemic inflammation, extending treatment-period microecologic changes from local intestinal injury to systemic metabolic regulation. Persistent dysbiosis and bile-acid imbalance may reduce tolerance of subsequent cycles and aggravate gastrointestinal and metabolic toxicities; their direct relationship with acquired paclitaxel resistance in breast-cancer cells still requires longitudinal patient samples and functional experiments (**Figures 3A, C**).

## 5 Gut and local microbiome regulation of chemotherapy response in gynecologic cancers

Ovarian-cancer evidence predominantly concerns the intestinal microbiota and its systemic metabolic and immune effects during platinum-based treatment (84, 96). Cervical-cancer studies include both gut microbiota and local cervical or tumor-associated microbial communities (85, 97). In endometrial cancer, chemotherapy-specific microbiome evidence remains limited, and available studies mainly characterize disease-associated gut microbial features (111).

### 5.1 Ovarian cancer

#### 5.1.1 Antibiotic exposure and microbiota dynamics during treatment

Integrity of the microbiota during treatment in patients with ovarian cancer is closely associated with outcomes of platinum-based chemotherapy. One study included 424 patients with newly diagnosed stage III–IV epithelial ovarian cancer, all of whom received platinum and taxane chemotherapy after cytoreductive surgery. Of these patients, 147 received antibiotics for more than 48 hours during chemotherapy, including 48 who received agents targeting Gram-positive bacteria. Compared with patients without antibiotic use, median progression-free survival in all antibiotic-exposed patients decreased from 23.1 to 17.4 months, with an adjusted hazard ratio (HR) of 1.31 (95% confidence interval [CI], 1.04–1.65); median overall survival decreased from 62.4 to 45.6 months, with an adjusted HR of 1.52 (95% CI, 1.18–1.96). Median progression-free and overall survival in patients exposed to agents targeting Gram-positive bacteria were 16.5 and 35.0 months, respectively, showing a stronger association (112). Infection, postoperative complications, and indications for antibiotic use may contribute to this association; overall, these data link antibiotic exposure and likely disruption of commensal microbiota with poorer long-term outcomes during platinum-combination chemotherapy (112).

Longitudinal microbial changes reflect the trajectory of platinum treatment in ovarian cancer better than a single baseline characteristic. D’Amico et al. serially collected fecal samples from 24 patients with epithelial ovarian cancer during neoadjuvant or adjuvant chemotherapy, obtaining 406 samples; 12 patients each received neoadjuvant and adjuvant treatment, and ultimately 9 developed platinum resistance whereas 15 remained platinum-sensitive. A single sample at diagnosis did not stably distinguish the two outcomes, but patients with platinum resistance showed progressive declines in α-diversity, loss of individual microbial fingerprints, and greater community fluctuations during treatment, with enrichment of taxa associated with lactate production such as *Coriobacteriaceae*, *Eggerthella*, and *Bifidobacterium*. Microbiota in platinum-sensitive patients were relatively stable and enriched for *Veillonellaceae*, which can utilize lactate (96). These longitudinal patterns associate greater ecological stability and enrichment of lactate-utilizing taxa with platinum-sensitive trajectories, whereas progressive diversity loss and enrichment of lactate-producing taxa characterize platinum-resistant trajectories (**Figure 3A**; **Table 3A**).

Serial sampling showed distinct intestinal ecological shifts after surgery and across successive chemotherapy cycles. Tong et al. obtained 75 fecal samples from 18 patients with ovarian cancer, including 10 preoperative samples, 4 postoperative prechemotherapy samples, and 61 samples after the first through fifth cycles of chemotherapy. Patients received carboplatin at AUC 6–7.5 plus paclitaxel 175 mg/m^2^ or cisplatin 75 mg/m^2^ plus paclitaxel 135 mg/m^2^, on a 21-day cycle. After radical surgery, relative abundances of *Bacteroidetes* and *Firmicutes* decreased whereas *Proteobacteria* increased; after entry into chemotherapy, *Proteobacteria* decreased from 64.50% before chemotherapy to 6.41%–18.77% during the cycles, whereas *Firmicutes* increased from 31.64% to 64.05%–78.70%, and anaerobes such as *Bacteroides*, *Collinsella*, and *Blautia* gradually increased with treatment cycles (113).

#### 5.1.2 Microbiota disturbance and metabolite-mediated cisplatin response

Animal studies support the notion that gut microbiota affect cisplatin efficacy by regulating tumor DNA damage, angiogenesis, and cancer stem cell status. Chambers et al. evaluated the effects of microbiota disturbance in immunocompetent C57BL/6J mice and immunodeficient mice using ID8 and ID8-VEGF syngeneic models and OV81 patient-derived xenografts, respectively. A broad-spectrum antibiotic combination of ampicillin, neomycin, metronidazole, and vancomycin was started before tumor inoculation and continued throughout cisplatin treatment. In the ID8-VEGF model, median survival was only 34 days in the group receiving antibiotics plus cisplatin, compared with 53 days in the group receiving control water plus cisplatin. Microbiota disruption was accompanied by attenuated cisplatin tumor inhibition, reduced tumor DNA-damage markers, enhanced angiogenesis, and increased cancer stem cells after chemotherapy. Transplantation of cecal microbiota from undisturbed donors into antibiotic-treated recipients partially restored cisplatin sensitivity and survival, and circulating indole-3-propionic acid, indoxyl sulfate, and hippuric acid also increased with microbiota reconstruction (84). Transfer of the microbial phenotype and rescue after transplantation indicate that commensal microbiota and their indole-related metabolites can maintain cisplatin-induced tumor-damage responses.

Different patterns of microbiota disturbance may also generate opposite metabolic outputs. Mai et al. found that antibiotic treatment enhanced cisplatin-induced tumor-cell apoptosis, while untargeted metabolomics showed significant enrichment of 3-methylxanthine in cecal contents after microbiota disruption. This association linked 3-methylxanthine to the microbiota-perturbation phenotype, and exogenous 3-methylxanthine further enhanced cisplatin effects both *in vivo* and *in vitro*, whereas intervention related to the dopamine D1 receptor weakened this sensitizing effect (114). The add-back experiment establishes the biological sufficiency of 3-methylxanthine to reproduce part of the sensitization phenotype under the tested conditions. Defining its endogenous microbiome contribution will require source-resolved measurements linking microbial production with systemic and tumor exposure.

#### 5.1.3 Gut microbiota and chemotherapy-related toxicity

The gut microbiome can also indirectly regulate ovarian-cancer outcomes by affecting chemotherapy-induced nausea and vomiting and treatment completion. Zu et al. collected feces before chemotherapy from patients with ovarian cancer receiving carboplatin or cisplatin plus paclitaxel for the first time and divided them into a chemotherapy-induced nausea and vomiting (CINV) group and a non-CINV group according to events after treatment, with 25 patients in each group. The CINV group was enriched for *Bacteroides caccae*, *Corynebacteriales*, and *Corynebacterium*; metabolomics identified 19 increased metabolites and suggested abnormal pentose-phosphate, glutathione, and lipoic-acid metabolism. The investigators further established a cisplatin-induced pica model in SD rats, with 10 rats per group. After transplantation of microbiota from patients without CINV, kaolin intake in rats decreased, and increases in 5-HT3R, NK1R, and NK2R in the medulla oblongata and colon were partially reversed (102) (**Figures 3B, C**; **Table 3B**).

The patient cohort identified baseline microbial and metabolic features associated with CINV, whereas microbiota-transfer experiments in rats support a gut–brain neurotransmitter-receptor pathway contributing to this toxicity phenotype (102). Persistent or severe CINV can lead to chemotherapy delay, dose reduction, or premature discontinuation, thereby reducing the delivered dose intensity of platinum and taxane therapy. Toxicity-associated microbiota may therefore be clinically relevant to treatment tolerance, delivered dose intensity, and chemotherapy completion.

### 5.2 Cervical cancer

Evidence in cervical cancer arises from two spatially distinct microbial compartments. Gut microbiota are associated primarily with systemic immunity, intestinal homeostasis, and treatment tolerance, whereas local cervical or tumor-associated microbiota can interact directly with the tumor microenvironment and modify tumor-cell responses to chemoradiotherapy. These compartments provide complementary, compartment-specific explanations for variation in treatment response and therefore need to be considered separately when interpreting microbiome–chemotherapy associations in cervical cancer.

#### 5.2.1 Gut-microbiota characteristics, remodeling during treatment, and chemoradiotherapy outcomes

Baseline gut-microbiota diversity in patients with cervical cancer is associated with immune activation and long-term survival after concurrent chemoradiotherapy. One study enrolled 55 patients with locally advanced cervical cancer who received 5 weeks of external-beam irradiation, weekly cisplatin, and subsequent brachytherapy. Multivariable analysis showed that higher baseline Shannon diversity was independently associated with longer recurrence-free survival, with an HR of 0.36 (95% CI, 0.15–0.84; P = 0.018), and was also associated with longer overall survival, with an HR of 0.19 (95% CI, 0.043–0.83; P = 0.028). *Porphyromonas*, *Porphyromonadaceae*, and *Dialister* were relatively enriched in short-term survivors, whereas *Escherichia-Shigella*, *Enterobacteriaceae*, and *Enterobacteriales* were relatively enriched in long-term survivors. Higher baseline diversity was also associated with increased CD4^+^CD69^+^ and CD4^+^PD-1^+^ T cells at week 3 and CD4^+^Ki67^+^ T cells at week 5; the latter two had Q values adjusted for multiple testing of 0.21 and 0.07, respectively (97). These results indicate that higher baseline microbial diversity is associated with a more favorable systemic immune profile during concurrent cisplatin chemoradiotherapy, including greater T cell activation and proliferative capacity. Because radiotherapy and cisplatin act concurrently, the survival signals are more likely to reflect host response to the overall chemoradiotherapy regimen than cisplatin sensitivity alone (97).

Longitudinal studies during treatment further show that concurrent chemoradiotherapy is accompanied by a progressive reduction in gut-microbiota diversity in parallel with worsening gastrointestinal function. A study of 35 patients with locally advanced cervical cancer collected samples at baseline and weeks 1, 3, and 5 of treatment. Microbial diversity declined continuously during the treatment course, most markedly at week 5; EPIC intestinal-function scores worsened concurrently, and higher diversity at each time point was associated with milder gastrointestinal symptoms, although baseline diversity did not stably predict toxicity at the end of treatment (103). Maintenance of microbial ecological stability during treatment may be closer to the patient’s real-time intestinal tolerance state than a single baseline measurement (**Figures 3A, B**; **Table 3B**).

Another study enrolled 58 patients receiving pelvic concurrent chemoradiotherapy, including 55 with cervical cancer, 2 with vulvar cancer, and 1 with vaginal cancer. All patients received at least 45 Gy in 25 fractions of external-beam irradiation together with weekly cisplatin at 40 mg/m^2^, and rectal swabs were collected at baseline and weeks 1, 3, 5, and 12. The mean Shannon index decreased from 2.91 to 2.52 during treatment (P < 0.01), accompanied by increased *Proteobacteria* and decreased *Clostridiales*. By week 12, microbial diversity in approximately 60% of patients had recovered close to baseline, but overall community structure had not completely recovered. A total of 42 patients used antibiotics during the study, so observed microbial changes were influenced by radiotherapy, cisplatin, and antimicrobial drugs (115). Persistent Proteobacteria expansion and Clostridiales depletion were observed alongside incomplete ecological recovery and gastrointestinal dysfunction, a pattern consistent with reduced SCFA-producing capacity and impaired epithelial-barrier recovery. Residual post-treatment community changes were likewise associated with ongoing gastrointestinal symptoms and alterations in immune status (115).

#### 5.2.2 Local cervical and tumor-associated microbiota in chemoradiotherapy response

Local cervical and tumor-associated microbiota constitute a spatially distinct microbial compartment that can directly influence tumor metabolism and cell survival during chemoradiotherapy. In an expanded set of 96 baseline cervical-tumor samples, Colbert et al. found that the presence of intratumoral *Lactobacillus iners* was associated with shorter recurrence-free survival; its relative abundance had an HR of 3.7 (95% CI, 1.0–13.3) for recurrence-free survival and an HR of 10.4 (95% CI, 1.8–60.3) for overall survival. The investigators isolated *L. iners* from cervical tumors and applied its sterile culture supernatant to patient-derived B1188 organoids and primary cervical cancer cells. *L. iners* produced substantially more L-lactate than D-lactate, and 20 mM L-lactate reproduced resistance of tumor cells to 4-Gy irradiation and gemcitabine, whereas its effects on cisplatin and 5-FU varied with cellular background. Transcriptomic and metabolomic analyses showed reprogramming of GLUT1, MCT1/MCT4, redox homeostasis, and lactate-utilization networks (85). *L. iners*-derived L-lactate can act as a metabolic substrate and signaling molecule to promote tumor-cell lactate uptake, energy utilization, and redox adaptation, thereby increasing survival under radiation and some chemotherapeutic drugs. These findings identify tumor-resident *L. iners* and its lactate output as a local metabolic pathway capable of modifying treatment response within the cervical tumor niche (85) (**Table 2**).

Composition of local microbiota and their evolution after treatment are also affected by host immune status. A Botswana cohort prospectively enrolled 53 patients with nonmetastatic stage IB2–IVA cervical cancer, all of whom received definitive radiotherapy; approximately 81% also received concurrent cisplatin. Forty-two patients had HIV infection and 11 were HIV-negative. The study used swabs from the cervical tumor surface for 16S rRNA sequencing. Pielou evenness was lower in patients with HIV infection than in HIV-negative patients (0.6 vs. 0.7, P = 0.02), with enrichment of *Parvimonas*, *Corynebacteriaceae*, and *Micrococcaceae*. Among the 42 patients with HIV infection, overall α- and β-diversity were relatively stable before and after treatment, but *Lactobacillaceae* and *Sutterella* were enriched before treatment, whereas *Ruminococcus*, *Caulobacterales*, and *Flavobacteriia* increased after treatment; higher post-treatment *Flavobacteriia* abundance was independently associated with poorer overall survival (116). Within this cohort, HIV status was associated with distinct local microbial composition and treatment-period trajectories, linking host immune status with local microecologic adaptation during chemoradiotherapy.

Because these samples were obtained from cervical-tumor surfaces, the observed changes primarily represent local microecologic trajectories. Together with the gut-microbiota evidence above, these findings indicate that cervical-cancer treatment response may reflect two spatially distinct microbial influences: gut-derived systemic immune and tolerance signals and direct local metabolic effects within the cervical tumor niche.

### 5.3 Endometrial cancer: emerging evidence and current gaps

Chemotherapy-specific microbiome evidence in endometrial cancer remains sparse. High-risk or recurrent disease is commonly treated with carboplatin plus paclitaxel, but currently available microbiome studies have largely characterized disease-associated microbial features rather than stratifying patients according to chemotherapy response. Moreover, obesity, diabetes, altered dietary patterns, and metformin exposure—all common in this population—can independently influence the gut microbiome (117). A metagenomic study including 15 patients with endometrial cancer and 15 healthy controls showed relatively increased *Klebsiella* and relatively decreased *Alistipes*, *Anaerobutyricum*, and *Oscillospira* in patients. *Klebsiella* enrichment may be associated with a proinflammatory microecology and impaired barrier function, whereas reductions in *Alistipes*, *Anaerobutyricum*, and *Oscillospira* may accompany decreased SCFA production and metabolic homeostasis (111). Because this study did not stratify patients by response to carboplatin plus paclitaxel treatment and did not conduct serial sampling during treatment, these microbial characteristics mainly reflect the intestinal ecological state associated with endometrial cancer and its metabolic background.

Microecologic studies of chemotherapy for endometrial cancer need to incorporate obesity, diabetes, metformin, antibiotics, and dietary variables into a unified analysis, and to combine serial sampling before treatment, during chemotherapy, and at disease progression to evaluate relationships between microbial function and carboplatin exposure, paclitaxel dose intensity, and objective response. Independent chemotherapy-specific cohorts and functional-validation models remain scarce, and translation from disease-associated microbiota to treatment-response mechanisms remains at an early stage.

## 6 Regulatory roles of the gut microbiome in chemotherapy response in genitourinary tumors

Genitourinary tumors differ markedly in their dependence on cytotoxic chemotherapy. Docetaxel remains used in metastatic castration-resistant prostate cancer (118). Muscle-invasive bladder cancer commonly receives cisplatin-based neoadjuvant chemotherapy (40). In contrast, clear-cell renal cell carcinoma is generally insensitive to conventional cytotoxic chemotherapy (119). The gut microbiome can influence these treatments through intestinal-barrier and inflammatory signals, systemic immunity, drug metabolism, and organ toxicity, with its effects depending on cancer type, chemotherapy regimen, and the ecological niche occupied by the microorganism (**Table 2**).

### 6.1 Prostate cancer

#### 6.1.1 Gut dysbiosis and prostate cancer progression

Prostate-cancer progression is closely associated with intestinal *Proteobacteria* expansion and systemic inflammatory status. Zhong et al. enrolled 35 participants, including 20 with benign prostatic hyperplasia, 10 with nonmetastatic prostate cancer, and 5 with metastatic prostate cancer. α-Diversity and β-diversity did not differ significantly among the three groups, but relative *Proteobacteria* abundance was markedly increased in patients with metastatic prostate cancer and was positively correlated with plasma IL-6 levels, regional lymph-node metastasis, and distant metastasis. *Proteobacteria* abundance could not distinguish prostate cancer from benign prostatic hyperplasia but could distinguish metastatic from nonmetastatic prostate cancer; its area under the receiver operating characteristic curve for predicting distant metastasis was 0.860 (P < 0.001), exceeding the discriminatory performance of concurrent PSA (86).

*Proteobacteria* enrichment is commonly accompanied by increased lipopolysaccharide burden, impaired intestinal-barrier function, and low-grade systemic inflammation. The observed associations among Proteobacteria enrichment, increased IL-6, and metastatic disease provide clinical context for the LPS-related inflammatory pathway that is tested directly in the subsequent docetaxel models (86).

#### 6.1.2 Dysbiosis weakens docetaxel efficacy through LPS/STAT3 signaling

Microbiota-transfer and pathway-inhibition experiments further reveal the causal role of gut microecology in docetaxel sensitivity. Investigators treated C57BL/6J mice with ampicillin, colistin, neomycin, and vancomycin and subsequently established subcutaneous and orthotopic RM-1 prostate-cancer models. Antibiotic treatment caused relative *Proteobacteria* enrichment, increased intestinal permeability and intratumoral LPS, and increased tumor volume, tumor weight, and Ki-67 expression. After feces from dysbiotic donors were transplanted into recipients that had not received antibiotics, the tumor-promoting phenotype was also transferred (86).

Docetaxel markedly inhibits tumor growth in mice with normal microbiota, but its tumor-inhibitory effect is clearly weakened in dysbiotic mice. Increased intratumoral LPS is accompanied by NF-κB activation, increased IL-6 expression, and enhanced STAT3 phosphorylation, indicating that gut-derived inflammatory signals can raise the survival threshold of tumor cells. When the STAT3 inhibitor Stattic at 2.5 mg/kg was combined with docetaxel at 10 mg/kg, tumor response to docetaxel was partially restored, forming a continuous regulatory sequence in which dysbiosis promotes intestinal-barrier leakage and circulating LPS, activates NF-κB/IL-6/STAT3 signaling, and enhances antiapoptotic survival (86).

Experiments in RM-1 and DU145 cells further support STAT3-mediated antiapoptotic effects. Relevant conditioned-medium treatment increases p-STAT3, Bcl-2, and Survivin expression and reduces docetaxel-induced apoptosis; after Stattic is added, tumor-cell viability decreases and TUNEL-positive cells increase (86). The *Proteobacteria*–IL-6 association in patient samples, phenotype transfer following fecal microbiota transplantation, and restoration of drug efficacy after STAT3 inhibition together support involvement of a gut-derived LPS/IL-6/STAT3 axis in docetaxel resistance in prostate cancer (86) (**Figure 4B**; **Table 2**).

### 6.2 Urothelial carcinoma

#### 6.2.1 Gut-microbiota characteristics, treatment-period dynamics, and response to neoadjuvant chemotherapy

After cisplatin-based neoadjuvant chemotherapy for muscle-invasive bladder cancer, pathological complete response or substantial downstaging is usually associated with a lower risk of postoperative recurrence (120). Response-related information in the gut microbiota is mainly reflected by functional combinations of multiple taxa rather than a single dominant genus. A multicenter prospective study of urothelial carcinoma enrolled 142 patients with bladder cancer and 48 healthy controls (13). Compared with controls, patients with bladder cancer had relative increases in intestinal *Prevotella* and *Porphyromonas* and a decrease in *Faecalibacterium*; however, overall community types generated by hierarchical clustering did not stably distinguish response to neoadjuvant chemotherapy (13). Among 57 fecal samples with complete efficacy information, a microbial-feature model predicted complete response with an area under the curve of 0.88 (95% CI, 0.81–0.94), markedly exceeding the value of 0.50 for a clinical-variable model; sensitivity and specificity were 0.70 and 0.80, respectively. *Oscillibacter*, *Fusicatenibacter*, and *Bacteroides* contributed strongly to the model, but no single genus independently dominated prediction (13). Total fecal SCFA concentrations were not significantly associated with chemotherapy response, although some microbiota were associated with specific metabolites. *Akkermansia* and *Clostridia* were associated with isobutyrate levels, whereas *Lactobacillus* and *Enterobacteriaceae* were associated with propionate levels (13). These findings suggest that microecologic signals associated with chemotherapy response may be jointly constituted by specific microbial combinations, metabolic functions, and host background, with greater information density than a single diversity or metabolite indicator (**Table 3A**).

Longitudinal sampling during cisplatin-containing treatment revealed changes in specific microbial taxa without a marked decline in overall diversity. The study collected fecal samples before treatment, after cycle 2 of dose-dense methotrexate/vinblastine/doxorubicin/cisplatin, and before cystectomy, and included cohabiting partners to control for shared diet and living environment. α-Diversity and β-diversity remained generally stable during neoadjuvant chemotherapy, but *Bacteroides*, *Escherichia-Shigella*, and *Roseburia* increased after treatment, whereas similar changes were not observed in cohabiting partners (13). Thus, changes in specific functional groups and their stability under chemotherapy pressure may more closely reflect drug response than overall community diversity. However, the microbial prediction model was actually developed from 57 patients with complete efficacy information; external multicenter validation and stratification by treatment regimen will determine whether the model can be translated into a stable clinical-stratification tool (13).

#### 6.2.2 Gut-systemic immune sensitization and local intratumoral drug inactivation

Available experimental studies suggest that microorganisms can affect the therapeutic action of gemcitabine plus cisplatin (GC) through two directions—systemic immune regulation and local drug metabolism—although their evidence sources and ecological niches differ substantially. With respect to systemic immune sensitization, commensals can amplify the tumor-inhibitory effect of GC by enhancing antigen presentation and cytotoxic immune responses. In an MBT2 urothelial-carcinoma model, 5-week-old male C3H mice were subcutaneously inoculated with 2 × 10^5^ tumor cells and randomly assigned to eight groups (121). GC altered intestinal community composition, and after oral administration of *Lactobacillus casei* strain Shirota and *Bifidobacterium breve*, the combined-intervention group already showed more pronounced tumor-growth inhibition at treatment week 2 (121). Treatment enhancement was accompanied by increased dendritic cell maturation and CD8^+^ T cell activation and reduced recruitment of Tregs and cancer-associated fibroblasts. Dendritic cell activation can increase the efficiency of tumor-antigen presentation, whereas increased CD8^+^ T cells facilitate clearance of tumor cells damaged by gemcitabine and cisplatin; reductions in Tregs and cancer-associated fibroblasts can also alleviate immunosuppression and the stromal barrier (121). The resulting pathway linking commensals, dendritic cells, CD8^+^ T cells, and tumor-microenvironment remodeling can amplify the initial tumor damage caused by GC without directly altering platinum–DNA adducts (**Figure 4D**; **Table 2**).

A distinct local mechanism is intratumoral bacterial inactivation of gemcitabine. In pancreatic ductal adenocarcinoma, Geller et al. showed that intratumoral Gammaproteobacteria express the long isoform of cytidine deaminase, which converts gemcitabine into inactive 2′,2′-difluorodeoxyuridine. Fourteen of 15 tumor-isolated bacterial strains reduced gemcitabine activity *in vitro*, whereas ciprofloxacin restored treatment efficacy in mouse models (9). These experiments establish bacterial cytidine deaminase as a mechanism capable of reducing local gemcitabine activity. In urothelial carcinoma, where gemcitabine forms part of standard GC chemotherapy, this cross-cancer mechanism identifies intratumoral bacterial cytidine deaminase as a biologically plausible local resistance node that may reduce effective gemcitabine activity within the tumor. Its relevance to urothelial carcinoma can be further defined by integrating intratumoral Gammaproteobacteria, cytidine-deaminase activity, gemcitabine/dFdU levels, and treatment response (**Figure 4A**; **Table 2**).

## 7 Predictive value of microbiota-associated and metabolic biomarkers in chemotherapy

### 7.1 Microbiome features and metabolic biomarkers for CRC chemotherapy

Clinical studies have increasingly evaluated whether microbial signatures can be measured reproducibly and used to predict chemotherapy response in CRC. Among individual taxa, *F. nucleatum* has the most substantial clinical evidence. In a retrospective cohort of 280 patients with stage III/IV CRC, a high intratumoral *F. nucleatum* burden was associated with invasion, lymph-node and distant metastasis, and reduced disease-free survival benefit from adjuvant chemotherapy; its tissue abundance therefore represents a candidate adverse prognostic and treatment-response marker (18). *B. fragilis* enrichment has likewise been observed in chemotherapy nonresponders and associated with poor survival, supporting its evaluation as a candidate response biomarker in prospective regimen-stratified cohorts (19). CoPEC colonization was associated with lower overall survival in stage III–IV CRC (20), whereas *F. prausnitzii* and *A. muciniphila* were more abundant in chemotherapy-sensitive patients (122). At the community level, tumor-tissue profiling identified lower microbial diversity in resistant disease and a six-genus signature—*Roseburia*, *Helicobacter*, Gardnerella, Flavonifractor, *Coprococcus*, and *Anaerostipes*—associated with recurrence-free survival (66). In a small exploratory cohort of 17 patients with metastatic CRC, responders were enriched in *Lactobacillus*, the nonprogressive disease group showed greater *Bifidobacterium abundance*, and community composition varied with RAS status (123). These findings support the evaluation of multi-taxon panels rather than a single universal microbial marker. Before clinical implementation, these signatures require external prospective validation with prespecified endpoints, standardized sampling and quantification, and clinically defined cutoffs. Analyses should also account for major clinical and microbiome-related confounders, including treatment regimen, antibiotic exposure, and diet (3, 124) (**Figure 1**; **Table 1**).

Metabolic biomarkers considered within a gut-microbiome framework require careful attribution of their biological origin. Fecal and circulating metabolite profiles may reflect microbial metabolism, host metabolism, dietary exposure, or shared host–microbial metabolic pathways. Accordingly, metabolites are described here as microbiota-derived only when microbial production is supported by functional, intervention, or other source-resolving evidence. Metabolites that correlate with microbial features without definitive source attribution are referred to as microbiota-associated, whereas analytes identified solely through serum or plasma metabolomics are described as circulating metabolic biomarkers. This distinction is particularly relevant to SCFAs, tryptophan-related metabolites, polyamines, and markers related to bile acids, whose biological sources and measured compartments differ substantially across studies.

Recent studies have evaluated circulating and fecal metabolite profiles associated with the gut microbiota as potential indicators of treatment response. Their interpretation, however, depends on the biological compartment sampled and the timing of collection. For SCFAs, resistant tumor tissues were enriched in *Roseburia and* Fusobacteria, and one study proposed fecal butyrate as a candidate response marker (66). However, local intratumoral exposure and fecal concentration are not interchangeable; the direction of association and the decision threshold should therefore be validated separately for each sample matrix. In patients with advanced CRC receiving neoadjuvant chemoradiotherapy, favorable response was associated with enrichment of microbial metabolic genes and evidence implicating GDP-D-rhamnose and butyrate (125). Responders in another cohort showed enrichment of butyrate-producing bacteria and higher fecal acetate, butyrate, isobutyrate, and hexanoate levels, whereas nonresponders had higher serum spermine and acetylated polyamine levels (126). The fecal SCFA profile was accompanied by microbial compositional differences, whereas the circulating polyamine signal identified in serum supports its role as a metabolic response marker, while attribution of its microbial contribution depends on source-resolved evidence. These findings favor compartment-resolved interpretation of metabolic biomarkers rather than reliance on fecal butyrate as a stand-alone marker. For tryptophan metabolism, an increase in circulating indole-3-propionic acid (IPA) after chemotherapy was associated with lower monocyte levels and was proposed as a dynamic response marker (104); diet-dependent effects and the shift toward kynurenine and away from indole metabolites in stage IV CRC further indicate that baseline context matters (72, 127). Kynurenine in this setting predominantly reflects host IDO1-mediated tryptophan metabolism, whereas IPA and related indoles have stronger evidence for microbial production. These analytes should therefore be considered biologically distinct components rather than grouped uniformly as microbiota-derived metabolites, and microbial attribution should be supported by paired metagenomics, functional profiling, or other source-resolving evidence. As circulating metabolic markers, serum spermine and acetylated polyamines discriminated neoadjuvant-chemoradiotherapy nonresponders with an area under the curve (AUC) of 0.718 (126), while a high PAMscore was associated with advanced disease and immunosuppressive features (23). A 12-gene bile acid metabolism score derived from The Cancer Genome Atlas (TCGA) data predicted prognosis and differential oxaliplatin sensitivity in an external dataset (128), but this transcriptomic score should be distinguished from direct measurement of microbial bile acids. Overall, prospective testing should specify the sample matrix, collection time point, analytic platform, decision threshold, treatment regimen, and added value beyond established clinicopathological variables (**Figure 2**).

Beyond the classic predictive markers described above, Ling et al. systematically evaluated the associations of serum S100A12 and soluble CD14 (sCD14) levels with gut dysbiosis and chemotherapy response in a retrospective analysis of 104 patients with advanced CRC and 104 healthy controls. Serum S100A12 and sCD14 levels were significantly higher in patients with CRC than in healthy controls, with the most pronounced increases in patients with dysbiosis (30). Correlation analysis showed that S100A12 and sCD14 levels were positively correlated with the abundances of *F. nucleatum* and *Prevotella* and negatively correlated with the abundances of *Faecalibacterium prausnitzii* and *A. muciniphila*. The levels of both markers decreased significantly after chemotherapy, and baseline S100A12 and sCD14 levels were significantly higher in chemotherapy nonresponders than in responders. Receiver operating characteristic (ROC) curve analysis showed that their combined diagnostic accuracy was superior to that of either marker alone. This study indicates that serum S100A12 and sCD14 levels are closely associated with gut dysbiosis and chemotherapy response and may serve as promising noninvasive biomarkers for predicting the efficacy of CRC chemotherapy (30). In older patients with CRC, elevated serum S100A12 and soluble receptor for advanced glycation end products (sRAGE) reflect gut dysbiosis and systemic inflammation driven by IL-6/TNF-α, and their combination predicts chemotherapy failure with an AUC as high as 0.914 (129). These circulating proteins are host inflammatory biomarkers associated with gut dysbiosis rather than microbiota-derived products, and their predictive value is therefore better interpreted as reflecting the host–microbiome inflammatory interface.

At the metabolite level, the prospective single-center CRC-Tsen study performed untargeted serum and fecal metabolomic profiling in 33 patients with metastatic CRC (mCRC) receiving first-line systemic therapy. Patients with disease progression showed higher baseline fecal levels of p-cresol and D-ribose, metabolites linked to microbial dysbiosis and genotoxic or xenobiotic stress, whereas patients with partial response or stable disease showed higher fecal fumaric acid and very-long-chain fatty acids, consistent with a more favorable immunometabolic profile (122). Baseline plasma L-proline and desmosterol were also enriched in patients with disease progression, suggesting increased protein catabolism and cholesterol biosynthesis, respectively (122) (**Table 1**). These circulating metabolites are therefore best regarded as exploratory metabolic correlates of treatment outcome, with their microbial contribution remaining source-unresolved.

### 7.2 Significance of microbiota-associated and metabolic biomarkers in chemotherapy for other cancers

Beyond CRC, clinical studies in ovarian, lung, breast, and cervical cancers have evaluated gut microbial features, microbiota-associated metabolic profiles, host–microbiome immune markers, and circulating metabolites as candidate predictors of chemotherapy efficacy and toxicity (130). These biomarker classes should be interpreted separately because an association with treatment response does not by itself establish microbial origin. Their predictive performance is further influenced by cancer type, treatment regimen, sample matrix, sampling time point, and endpoint definition (**Tables 3A,B**).

#### 7.2.1 Pretreatment microbial features and stratification of chemotherapy efficacy

Pretreatment microbial combinations in ovarian cancer have shown high discriminatory ability for platinum sensitivity. Gong et al. included 174 patients with ovarian cancer who underwent cytoreductive surgery and carboplatin plus paclitaxel treatment. Among them, 77 patients who relapsed within 6 months after completing chemotherapy were defined as platinum resistant, whereas 97 whose time to relapse exceeded 6 months were defined as platinum sensitive. Pretreatment 16S rRNA sequencing showed that both the Shannon and Simpson indices were higher in the platinum-resistant group than in the sensitive group (both P < 0.05), with relative enrichment of Proteobacteria in the platinum-resistant group and predominance of Firmicutes in the platinum-sensitive group. Among nine machine-learning algorithms, the random-forest model had the best discriminatory performance, with an AUC of 0.909, and *Angelakisella*, *Arenimonas*, and *Roseburia* constituted the principal feature set (95). The increased microbial diversity in this cohort mainly reflected expansion of resistance-associated taxa, indicating that the clinical meaning of diversity metrics depends on the specific community composition.

Circulating metabolite panels may complement microbiome-based treatment stratification by capturing integrated host–microbial metabolic states. Sun et al. enrolled 323 participants, including 122 in an untargeted-metabolomics discovery cohort and 201 in an ultra-performance liquid chromatography–tandem mass spectrometry (UPLC–MS/MS) targeted-quantification cohort. Among patients with non-small cell lung cancer receiving first-line pemetrexed plus cisplatin or carboplatin, tryptophan metabolism differed most markedly between the sensitive and resistant groups. Resistant patients exhibited increased kynurenine and decreased tryptophan and xanthurenic acid, and a random-forest model based on these three circulating metabolites achieved an AUC of 0.954 for predicting treatment response (90). These findings support a circulating metabolic response classifier comprising kynurenine, tryptophan, and xanthurenic acid. Because kynurenine metabolism integrates host indoleamine 2,3-dioxygenase/tryptophan 2,3-dioxygenase (IDO/TDO) activity, hepatic and renal function, diet, and microbial metabolism, its microbial contribution should be resolved using paired microbiome–metabolome analyses or other source-resolving approaches.

Studies in cervical cancer have attempted to incorporate both the gut microbiota and peripheral immune status into predictive systems. A study in western Mexico included 77 women: 27 treatment-naive patients with cervical cancer, 22 patients receiving radiotherapy combined with platinum-containing chemotherapy, and 28 healthy controls. After treatment, patients showed decreased Firmicutes and increased Proteobacteria, accompanied by reductions in *Faecalibacterium*, *Ruminococcus*, *Roseburia*, and *Akkermansia* and increases in *Escherichia–Shigella* and *Bacteroides*. Peripheral-blood NK cells simultaneously exhibited increased co-expression of inhibitory receptors, including PD-1, LAG-3, TIM-3, TIGIT, BTLA, and NKG2A. The *Escherichia*/*Ruminococcus* ratio and the frequency of PD-1^+^CD56^bright^ NK cells were integrated into dysbiosis and NK cell exhaustion scores, respectively, and both scores were associated with poorer survival (98). Combined detection of microbial and immune phenotypes can simultaneously reflect intestinal ecology and effector-cell function, but the cross-sectional design limits its capacity to prospectively predict individual treatment response (**Table 3A**).

#### 7.2.2 Early dynamic changes during treatment and efficacy monitoring

Early microbial changes after treatment initiation may more directly reflect an emerging drug response than baseline samples. Qiu et al. enrolled 41 patients with non-small cell lung cancer receiving concurrent chemoradiotherapy from two phase II clinical trials (NCT02573506 and NCT03006575) (91). A total of 102 fecal samples were collected before treatment, 2 weeks after treatment initiation, and at the end of treatment, and patients were divided into groups with long versus short progression-free survival (PFS) using an 11-month cutoff (91). Baseline samples did not provide stable outcome discrimination, whereas microbial features at 2 weeks after treatment showed clear discriminatory ability. The long-PFS group had higher Shannon and phylogenetic-diversity indices, with P values of 0.006 and 0.022, respectively; a random-forest model composed of *Lachnospiraceae NK4A136*, *UCG-003*, *Dorea*, the *Eubacterium hallii* group, the *E. siraeum* group, and members of Muribaculaceae achieved an AUC of 0.87 (91).

Multivariable analysis showed that phylogenetic diversity and *Dorea* abundance at 2 weeks after treatment remained independently associated with PFS, with HRs of 8.036 (P = 0.016) and 4.186 (P = 0.043), respectively. Patients with long PFS were enriched in functions related to fatty-acid metabolism and arginine synthesis, whereas those with short PFS were enriched in lipopolysaccharide-biosynthesis pathways (91). Two weeks after treatment initiation may constitute a microecological monitoring window that precedes conventional imaging assessment, but the combined exposure to radiotherapy and chemotherapy means that the model reflects overall treatment response rather than sensitivity to a single drug. The small sample size and internal modeling also limit direct clinical application of the risk thresholds.

The advantage of dynamic biomarkers is their ability to simultaneously capture drug exposure, mucosal injury, and changes in host immunity (91, 100). Stable application of such indicators requires sampling times to be fixed relative to the chemotherapy cycle and administration date and synchronized evaluation against imaging response, pathological regression, and survival endpoints. The magnitude of change between time points may reflect a patient’s treatment trajectory better than a single absolute abundance (**Figure 3**; **Table 3A**).

#### 7.2.3 Predictive value of gut-microbial features for chemotherapy toxicity

In breast cancer, the pretreatment gut microbiota has been used to predict hematologic and gastrointestinal toxicities. Nguyen et al. enrolled 301 patients with breast cancer receiving first-line chemotherapy, collected fecal samples before treatment, and performed shotgun metagenomic sequencing, using grade ≥3 hematologic and gastrointestinal toxicities as endpoints (99). Higher baseline α-diversity was associated with lower risks of severe hematologic toxicity and severe neutropenia; the multivariable odds ratio (OR) was 0.94 for both outcomes, with 95% CIs of 0.89–0.99 and P values of 0.048 and 0.016, respectively. Enrichment of Synergistota, Synergistia, and Synergistales was associated with a lower risk of neutropenia, whereas Firmicutes C, Negativicutes, Firmicutes I, Bacilli A, and Paenibacillales were associated with increased risk (99).

Gastrointestinal toxicity exhibits a different ecological pattern. Severe nausea and vomiting were associated with a higher Chao1 index, a specific β-diversity structure, enrichment of 20 members of Lachnospiraceae, Oscillospiraceae, or Ruminococcaceae, and enrichment of pathways related to the reductive tricarboxylic-acid cycle; *Odoribacter laneus* and the L-proline biosynthesis II pathway were associated with lower risk (99).

Longitudinal sampling studies have further revealed the temporal relationship between baseline ecological vulnerability and gastrointestinal symptoms during treatment. Otto-Dobos et al. collected fecal samples, blood, and symptom data from 59 patients with breast cancer before, during, and after chemotherapy. Lower baseline α-diversity and reduced *Faecalibacterium* were associated with more severe subsequent nausea, vomiting, or diarrhea; patients with lower baseline diversity exhibited greater changes in Bray–Curtis distance during treatment, and the magnitude of community shift was positively correlated with diarrhea severity (100). The ecological stability of the baseline microbiota may determine the intestine’s buffering capacity against chemotherapy stress and influence the symptom burden during treatment.

Taxane-associated peripheral neuropathy also exhibits detectable microbial and metabolite changes. Beales et al. enrolled 70 patients with early breast cancer receiving paclitaxel or docetaxel and collected fecal and blood samples before the first chemotherapy cycle and within 3 days before the final infusion (101). After treatment, numbness, tingling, and limitations in daily activities increased significantly, with an OR of 9.0 for symptom severity (95% CI 3.3–34.8, P < 0.001), accompanied by an increase in circulating neurofilament light chain. Although overall α-diversity did not change significantly, unweighted UniFrac β-diversity changed (P = 0.001), and the magnitude of community shift was associated with the severity of sensory neuropathy (101).

In patients who developed neuropathy, taxa including *Collinsella*, *Faecalibacterium*, *Blautia*, *Dorea*, and *Anaerostipes* decreased, accompanied by reductions in fecal acetate, butyrate, and isovalerate and increases in cholic acid, chenodeoxycholic acid, taurocholic acid, and ursodeoxycholic acid (101). Microbial shifts, metabolite alterations, patient-reported symptoms, and circulating neurofilament light chain constitute a multimodal combination of indicators with complementary information, but its predictive performance remains to be established in independent treatment cohorts (**Figures 3B, C**; **Table 3B**).

## 8 Microbiota-targeted interventions and emerging microbe-inspired adjunctive technologies

The intervention strategies discussed in this section fall into two conceptually distinct categories. Microbiota-targeted interventions, including fecal microbiota transplantation (FMT), probiotics or postbiotics, and strain-specific phage therapy, directly modify microbial community structure, function, or resistance-promoting microorganisms and may thereby influence chemotherapy efficacy or tolerance. In contrast, engineered microorganisms, phage-derived nanostructures, bacterial membranes, and other biomimetic delivery systems primarily exploit microbial components, tropism, or synthetic-biology principles for tumor targeting, drug delivery, immune modulation, or stromal remodeling. These approaches are discussed separately as emerging microbe-inspired adjunctive technologies, with their chemotherapy relevance defined according to their principal therapeutic function.

### 8.1 Fecal microbiota transplantation

Fecal microbiota transplantation (FMT) reconstructs microbial composition, metabolic function, and mucosal immune homeostasis by introducing an intact donor-derived microbial community into the recipient intestine. Compared with supplementation with a single strain, FMT can simultaneously repair chemotherapy-associated dysbiosis, barrier damage, and metabolic disturbances. Its efficacy depends on whether functional donor bacteria can establish stable colonization and restore microecological functions associated with drug sensitivity, antitumor immunity, and chemotherapy tolerance (131) (**Table 4A**).

**Table 4:** Representative microbiota-targeted interventions and emerging microbe-inspired adjunctive technologies relevant to chemotherapy across solid tumors.

A. Microbiota-targeted interventions.					
Strategy/representative approach	Cancer and chemotherapy context	Microbial target/compartment	Key chemotherapy-related finding	Evidence type	Reference
FMT + capecitabine	CRC; capecitabine	Global gut microbiota and antitumor immunity	Restored microbial diversity and increased CD8^+^ T cell/NK cell infiltration; enhanced capecitabine-associated tumor control	Preclinical CRC model	(132)
Cecal microbiota transfer	Ovarian cancer; cisplatin after antibiotic depletion	Gut microbiota and microbial metabolic function	Restored circulating indole-related metabolites and partially restored cisplatin sensitivity	Animal model with microbiota rescue	(84)
*L. casei* Shirota + *B. breve*	Urothelial cancer; gemcitabine/cisplatin	*Gut–systemic antitumor immunity*	Enhanced DC/CD8^+^ T cell responses and chemotherapy-associated tumor control	Preclinical model	(121)
Probiotic yogurt preparations	Breast cancer; 5-FU/capecitabine	*Gut microbiota and intestinal toxicity*	Reduced intestinal injury and treatment-related mortality while preserving antitumor activity	Animal model	(133)
Phage VA7	CRC; 5-FU/oxaliplatin	*B. fragilis*	Blocked the SusD/RagB/Notch1/EMT axis and restored chemotherapy sensitivity	CRC mouse model	(19)
Phage-guided nanoparticles	CRC; fluoropyrimidine/irinotecan-based chemotherapy	*F. nucleatum*	Reduced tumor-associated *F. nucleatum* and potentiated chemotherapy through bacteria-targeted delivery	Preclinical CRC model	(134)
Antimicrobial–chemotherapy nanoplatform	CRC; cisplatin or sequential antimicrobial/chemotherapy treatment	Intratumoral *F. nucleatum*	Reduced intratumoral *F. nucleatum* burden and improved antitumor response through coordinated bacterial control and chemotherapy	CRC animal model	(135, 136)

B. Emerging microbe-inspired adjunctive therapeutic technologies					
Strategy/representative approach	Cancer and chemotherapy context	Primary therapeutic function	Key chemotherapy-related finding	Evidence type	Reference
phi29 pRNA EGFR-aptamer–siXBP1 platform	Triple-negative breast cancer; doxorubicin	XBP1-targeted payload delivery and chemotherapy potentiation	Enhanced doxorubicin-induced tumor-cell killing and tumor suppression through XBP1-targeted delivery	*In vitro* and xenograft model	(137)
Intratumoral HysA-secreting *Salmonella*	Breast and pancreatic cancers; doxorubicin/gemcitabine	Stromal remodeling and chemotherapy penetration	Reduced stromal compression and improved intratumoral chemotherapy penetration	Animal models	(138)
Collagenase-delivering engineered *E. coli*	Breast cancer; doxorubicin	*Collagen remodeling and intratumoral drug distribution*	Increased intratumoral doxorubicin distribution and tumor control through collagen remodeling	Animal model	(139)
CRS-207 + pemetrexed/cisplatin	Malignant pleural mesothelioma	Tumor-directed immune activation with chemotherapy	Clinical activity and immune remodeling observed in combination with pemetrexed/cisplatin; evaluated in a single-arm phase Ib study	Phase Ib single-arm study	(140)
DOX/BCG-PPD nanosystem	Bladder cancer; doxorubicin	Local doxorubicin delivery and immune activation	Improved intravesical doxorubicin delivery and local chemo-immunotherapy activity	Preclinical bladder cancer model	(141)

CRC, colorectal cancer; FMT, fecal microbiota transplantation; 5-FU, 5-fluorouracil; Fn, Fusobacterium nucleatum; EcN, Escherichia coli Nissle 1917; OXA, oxaliplatin; DOX, doxorubicin; EGFR, epidermal growth factor receptor; XBP1, X-box binding protein 1; siXBP1, small interfering RNA targeting XBP1; Treg, regulatory T cell; GPX4, glutathione peroxidase 4; HysA, hyaluronidase A; HA, hyaluronic acid; ColG, collagenase G; BCG-PPD, Bacillus Calmette–Guérin purified protein derivative; NK, natural killer; PFS, progression-free survival; OS, overall survival.

#### 8.1.1 Regulation of CRC chemotherapy efficacy and the immune microenvironment by FMT

CRC animal models show that FMT can enhance the effects of chemotherapy by reconstructing intestinal ecology and antitumor immunity. In an AOM/DSS-induced CRC mouse model, Arshad et al. transplanted fecal microbial suspensions from healthy mice into the recipient intestine by rectal enema. They found that although capecitabine alone reduced the abundance of pathogenic bacteria, FMT supplementation reversed gut dysbiosis more effectively, and the ACE and Chao1 indices and principal coordinate analysis (PCoA) all confirmed significant restoration of microbial diversity. Specifically, FMT supplementation increased the proportions of CD4^+^ T cells, CD49b^+^ NK cells, CD8^+^ T cells, and Ly6G^+^ neutrophils, enhanced IL-10 and IFN-γ production, and suppressed multiple inflammatory factors more strongly than capecitabine alone (132). In another study, AOM/DSS-induced CRC mice received salinomycin combined with FMT. FMT supplementation significantly increased propionate and butyrate levels, promoted infiltration of CD8^+^ T cells and Ly6G^+^ neutrophils, and reduced recruitment of F4/80^+^ macrophages. Cytokines unaffected by salinomycin alone all exhibited strong responses to microbial changes after the addition of FMT (142).

By constructing four patient-derived CRC xenograft (PDX) models, Bielawska et al. systematically evaluated the effects of FMT on the gut microbiota, metabolites, and the efficacy of folinic acid, 5-fluorouracil, and oxaliplatin (FOLFOX) chemotherapy. They found that FMT markedly altered gut-microbial structure, with clear changes in both α- and β-diversity, but had minimal or almost no effect on fecal SCFA and amino-acid levels. This finding suggests that the chemosensitizing effect of FMT varies substantially among individuals and may not operate through the conventional SCFA pathway; rather, it may be achieved by direct regulation of the tumor microenvironment or host immune response through changes in microbial composition (143).

Regarding improvement of post-chemotherapy immune recovery by FMT, Yu et al. transplanted gut microbiota from healthy mice into CRC mice by rectal enema in an AOM/DSS-induced CRC mouse model. The severely disrupted gut microbiota in CRC mice was largely reversed by FMT, the diameter and number of cancerous lesions decreased significantly, intestinal mucosal architecture was restored, and survival was significantly prolonged. Large numbers of infiltrating CD8^+^ T cells and CD49b^+^ NK cells were present in the intestines of FMT-treated mice and were capable of directly killing tumor cells; meanwhile, immunosuppressive Foxp3^+^ Treg cells that had accumulated in CRC mice were significantly reduced after FMT. FMT also regulated inflammatory-cytokine expression, including downregulation of IL-1α, IL-6, IL-12α, IL-12β, and IL-17α and upregulation of IL-10 (33).

#### 8.1.2 Alleviation of chemotherapy-associated intestinal toxicity by FMT

Chemotherapy-associated mucosal damage not only limits patient tolerance but may also reduce actual dose intensity through dose reduction, delays, and treatment interruption. FMT can alleviate this toxicity by restoring barrier architecture and suppressing inflammatory signaling. In a model of FOLFOX-induced intestinal mucositis, chemotherapy caused diarrhea, goblet-cell depletion, reduced expression of the tight-junction protein ZO-1, increased intestinal-epithelial apoptosis, and activation of the TLR/MyD88/NF-κB pathway and IL-6-related inflammatory responses. Daily FMT alleviated diarrhea and histologic damage, restored microbial composition, suppressed TLR/MyD88/NF-κB signaling, and caused no observed bacteremia (144) (**Table 4A**).

During irinotecan treatment, gut-microbial β-glucuronidase can reactivate SN-38G, which has been detoxified by the liver and excreted into the intestine, into the toxic form SN-38, thereby aggravating delayed diarrhea, a dose-limiting toxicity. Using FMT from CRC patients with distinct fecal SN-38G metabotypes, Wu et al. showed that differences in microbial SN-38G hydrolysis activity and irinotecan-induced gastrointestinal toxicity could be transferred to pseudo-germ-free mice; inhibition of gut microbial β-glucuronidase attenuated toxicity in mice receiving microbiota from extensive metabolizers (145).

Notably, in a study of the toxic adverse effects of FMT, He et al. collected feces from mice without subcutaneous tumors that had been treated with high-dose oxaliplatin and transplanted them into healthy mice. FMT from oxaliplatin-exposed mice transferred chemotherapy toxicity to the recipients. Splenic RNA sequencing and macrophage-depletion experiments showed that microbially induced oxaliplatin toxicity depended on macrophages, and that the oxaliplatin-induced microbiota aggravated chemotherapy toxicity by suppressing IL-10 secretion from macrophages (146).

#### 8.1.3 Role of FMT in chemotherapy for other solid tumors

Models of other solid tumors further indicate that FMT can both restore chemotherapy sensitivity and transfer a low-response phenotype, with the direction determined by the functional state of the donor microbiota. In ovarian-cancer models, microbial disruption induced by broad-spectrum antibiotics impaired cisplatin efficacy, whereas replenishment with cecal microbiota from undisturbed donors partially restored cisplatin sensitivity and increased circulating levels of microbiota-associated metabolites such as indole-3-propionic acid, indoxyl sulfate, and hippuric acid (84).

Breast-cancer models show that FMT from donors fed a high-fat diet can transfer an unfavorable chemotherapy phenotype. This microbiota transplantation increases circulating LPS, weakens doxorubicin-induced tumor-cell apoptosis, and promotes tumor growth and lung metastasis; exogenous LPS reproduces similar changes (82). The donor’s dietary and metabolic-inflammatory state therefore emerges as an important determinant of whether FMT transfers favorable or unfavorable chemotherapy phenotypes, highlighting donor functional state as a key variable for clinical translation. These cross-tumor models provide a mechanistic basis for testing donor selection, engraftment, and treatment-response endpoints in clinical FMT studies.

#### 8.1.4 Clinical translation of FMT: human evidence, trial design, donor selection, and safety

Clinical translation of FMT in oncology is progressing from safety-focused studies toward therapy-directed protocols. In a prospective single-center study, Mendelsohn et al. evaluated FMT for recurrent *Clostridioides difficile* infection in 10 patients with solid tumors who had received chemotherapy within the preceding 6 months. No infectious complications attributable to FMT were observed, and 8 of 10 patients achieved cure after the first FMT. This study provides direct safety data in a chemotherapy-exposed population, although its clinical endpoint was treatment of recurrent *C. difficile* infection rather than modulation of antitumor efficacy (147). A more direct chemotherapy-oriented study is FMT-JSNO-02, a published protocol for a prospective multicenter single-arm exploratory trial designed to enroll 62 previously untreated patients with driver gene-negative advanced NSCLC receiving oral FMT capsules together with platinum-doublet chemotherapy and tislelizumab. The primary endpoint is the 12-month progression-free survival rate. As a prospective multimodal study, FMT-JSNO-02 can define the feasibility, safety, microbiome dynamics, and combination-level efficacy signals of integrating FMT with chemoimmunotherapy; quantification of the incremental contribution of FMT will require a controlled design (75) (**Table 5**).

**Table 5:** Clinical translation of FMT and probiotic interventions in oncology.

Intervention	Cancer/treatment setting	Study design and status	Primary clinical focus	Main finding or protocol feature	Translational relevance and design context	References
FMT	Solid tumors; chemotherapy-exposed patients with recurrent *C. difficile* infection	Prospective single-center observational study; n = 10; completed	FMT safety and CDI cure	No FMT-attributable infectious complications; 8/10 cured after first FMT	Establishes FMT safety and feasibility in chemotherapy-exposed patients; the clinical endpoint was treatment of recurrent CDI.	(147)
FMT	Advanced driver gene-negative NSCLC; platinum-doublet chemotherapy + tislelizumab	Prospective multicenter single-arm exploratory protocol; planned n = 62	12-month PFS; feasibility and safety	Oral FMT capsules administered with standard chemoimmunotherapy	Direct chemotherapy-relevant protocol evaluating feasibility, microbiome dynamics, and combination-level efficacy; incremental FMT effects require controlled comparison.	(75)
FMT	Metastatic melanoma refractory to anti-PD-1 therapy; FMT + anti-PD-1 reinduction	Phase I; n = 10; completed	Safety/feasibility and clinical response	Three clinical responses, including two partial and one complete response	Establishes oncology FMT feasibility and provides donor-selection and engraftment experience applicable to subsequent systemic-treatment trials.	(148)
FMT	Melanoma refractory to anti-PD-1 therapy; responder-derived FMT + anti-PD-1	Clinical trial; n = 15; completed	Safety, microbiome remodeling and clinical benefit	Clinical benefit in 6/15 patients with durable microbiome and immune changes	Demonstrates donor-derived microbiome remodeling and links engraftment with immune and clinical phenotypes, providing pharmacodynamic principles for future FMT trials.	(149)
FMT	Previously untreated advanced melanoma; healthy-donor FMT + nivolumab/pembrolizumab	Multicenter phase I; n = 20; completed	Safety	No grade 3 adverse events attributed to FMT alone	Provides first-line oncology FMT safety, donor-selection, and engraftment data relevant to future combinations with systemic anticancer therapy.	(150)
FMT	Treatment-naive metastatic RCC; selected-donor FMT + pembrolizumab/axitinib	Randomized, double-blind, placebo-controlled phase 2a; n = 45; completed	12-month PFS; secondary PFS, ORR, safety and microbiome changes	Primary endpoint did not reach significance; median PFS favored donor FMT and donor-strain engraftment was demonstrated	Provides a randomized controlled framework for evaluating donor-FMT engraftment and incremental treatment effects on background systemic therapy.	(151)
Probiotics	CRC; postoperative chemotherapy	Randomized placebo-controlled study; n = 100; completed	Gastrointestinal toxicity and microbiome changes	Reduced chemotherapy-associated gastrointestinal complications, particularly diarrhea; microbiome/SCFA changes observed	Supports a treatment-tolerance role for defined probiotics through mitigation of chemotherapy-associated gastrointestinal toxicity.	(152)
Probiotics	Locally advanced nasopharyngeal carcinoma; concurrent chemoradiotherapy	Randomized, double-blind, placebo-controlled trial; n = 99; completed	Severe oral mucositis	Reduced severe oral mucositis and attenuated treatment-associated T cell reduction	Supports mitigation of treatment-associated mucosal toxicity during concurrent chemoradiotherapy.	(153)
Probiotics	Stage I–III breast cancer; PLD-based adjuvant chemotherapy	Randomized placebo-controlled trial; completed	Hand–foot syndrome, oral mucositis, quality of life	Reduced incidence and severity of HFS and OM and altered microbial/metabolic features	Supports chemotherapy-tolerance benefit through reduction of hand–foot syndrome and oral mucositis.	(29)
Probiotics	Stage II–IV or platinum-sensitive recurrent high-grade ovarian cancer; platinum chemotherapy	Randomized, double-blind, placebo-controlled published protocol; planned n = 124	Primary: longitudinal gut-microbiome composition; secondary/exploratory clinical, toxicity, metabolic and immune endpoints	Defined five-strain formulation plus inulin administered across platinum-chemotherapy cycles	Provides a prospective framework integrating defined-strain probiotics, longitudinal microbiome profiling, toxicity, immune, metabolic, and clinical outcomes during platinum chemotherapy.	(154)

CDI, Clostridioides difficile infection; CRC, colorectal cancer; FMT, fecal microbiota transplantation; HFS, hand–foot syndrome; ICI, immune checkpoint inhibitor; NSCLC, non-small cell lung cancer; OM, oral mucositis; ORR, objective response rate; PFS, progression-free survival; PLD, pegylated liposomal doxorubicin; RCC, renal cell carcinoma; SCFA, short-chain fatty acid; TKI, tyrosine kinase inhibitor.9 Current challenges and future directions for microbiota-targeted and microbe-inspired strategies in chemotherapy.

Completed oncology FMT trials have so far concentrated predominantly on immune-checkpoint blockade rather than cytotoxic chemotherapy. In melanoma refractory to anti-PD-1 therapy, Baruch et al. conducted a phase I study in 10 patients and observed clinical responses in three patients after FMT followed by anti-PD-1 reinduction (148), whereas Davar et al. reported clinical benefit in 6 of 15 patients treated with responder-derived FMT plus anti-PD-1 therapy (149). Routy et al. subsequently evaluated healthy-donor FMT combined with nivolumab or pembrolizumab in a multicenter phase I study of 20 previously untreated patients with advanced melanoma; safety was the primary endpoint, and no grade 3 adverse events were attributed to FMT alone (150). More recently, the randomized, double-blind, placebo-controlled phase 2a TACITO trial evaluated selected-donor FMT in 45 treatment-naive patients with metastatic renal cell carcinoma receiving pembrolizumab plus axitinib. Although the primary 12-month progression-free survival endpoint did not reach statistical significance, the trial demonstrated donor-strain engraftment and provided a controlled framework for evaluating the incremental effect of FMT on background systemic therapy (151). Together, these studies illustrate the evolution of oncology FMT from safety and proof-of-concept cohorts toward controlled donor-FMT designs and provide a trial-design framework for chemotherapy-specific studies (**Table 5**).

Clinical use of FMT during chemotherapy requires particularly stringent donor qualification and product standardization because neutropenia and chemotherapy-induced mucosal-barrier injury may increase the consequences of transmitting opportunistic or antimicrobial-resistant organisms. Donor assessment should combine medical and exposure history with screening for enteric pathogens and multidrug-resistant organisms, while stool-bank workflows should provide source traceability, standardized collection, processing and storage, predefined release criteria, and longitudinal safety monitoring (155). The clinical relevance of these safeguards was illustrated by the documented transmission of extended-spectrum β-lactamase-producing *Escherichia coli* from a single FMT donor to two recipients, one of whom died (156). Donor selection should also extend beyond pathogen exclusion because strain engraftment varies substantially among recipients and disease settings and is influenced by recipient and treatment characteristics (157). Accordingly, chemotherapy trials should prospectively define donor-selection criteria, FMT dose and route, timing relative to cytotoxic treatment and antibiotics, engraftment endpoints, infection surveillance, and mechanism-matched clinical endpoints. From a regulatory and manufacturing perspective, clinical FMT programs require protocol-specific governance of donor eligibility, product traceability, processing and storage, lot-level release criteria, and adverse-event surveillance; regulatory requirements remain dependent on the intended use, product classification, and jurisdiction (155, 158).

### 8.2 Probiotics, postbiotics, prebiotics, and synbiotics

Probiotics regulate chemotherapy response by supplementing strains with specific metabolic or immune functions, whereas prebiotics promote the restoration of endogenous beneficial bacteria and their metabolic functions by providing selectively fermentable substrates. Compared with FMT, the composition and dose of both types of preparation are easier to control and more amenable to standardized production and repeated administration, but their effects are jointly influenced by strain characteristics, substrate type, the recipient microbiota, and the chemotherapy regimen (39). These interventions can both increase the drug sensitivity of tumor cells and improve chemotherapy outcomes by maintaining the mucosal barrier, regulating antitumor immunity, and alleviating treatment toxicity (159) (**Table 4A**).

#### 8.2.1 Regulation of CRC chemosensitivity by probiotics

Extracellular vesicles derived from *L. plantarum* preparations downregulate PDK2 expression through p53–p21-dependent metabolic signaling, reverse the enhanced glycolytic metabolism of 5-FU-resistant CRC cells, suppress cancer-cell proliferation, and ultimately induce apoptosis, thereby restoring the chemosensitivity of 5-FU-resistant cells (39). 5-FU-resistant CRC cells are insensitive to butyrate, primarily because expression of the butyrate transporter SMCT1 is downregulated. Metabolites derived from *L. plantarum* preparations restore functional SMCT1 expression through epigenetic or transcriptional regulation, thereby resensitizing CRC cells to butyrate and 5-FU (52). γ-aminobutyric acid (GABA)-producing *L. plantarum* preparations activate antiproliferative, antimigratory, and anti-invasive effects through GABA_B_ receptor signaling and induce apoptosis in 5-FU-resistant HT-29 cells (40).

A probiotic mixture containing *L. plantarum*, *L. brevis*, *L. helveticus*, and *L. delbrueckii*, used alone or in combination with 5-FU, effectively suppresses CRC tumor growth by regulating the apoptosis-related genes *Bax* and *Bcl-2*, downregulating collagen and TGF-β expression, suppressing the inflammatory factors TNF-α, IL-6, and IL-1β, and inhibiting cell migration through regulation of E-cadherin and MMP2 expression (160). In the AOM/DSS model, the novel probiotic mixture Prohep showed a stronger antitumorigenic effect than 5-FU alone or the combination treatment, significantly reducing total tumor number and size, cecal weight, colonic-crypt depth, colonic inflammation, and collagen fibrosis. Prohep also downregulated pro-inflammatory TNF-α and proliferative p-STAT3 and upregulated apoptotic p53. Metagenomic analysis showed that Prohep-enriched taxa, including *Helicobacter ganmani* and *Desulfovibrio porci*, were negatively correlated with total tumor number. Finally, Prohep also increased acetate concentrations and the biosynthesis of beneficial metabolites such as L-lysine, lipoic acid, pyrimidine, and palmitate (161).

With respect to *Lactobacillus fermentum* preparations, a mixture of *L. acidophilus* and *L. casei* increases the capacity of 5-FU to induce apoptosis, enhancing the apoptosis-inducing effect of 5-FU by 40% (162). Combined application of cellular extracts from *L. fermentum* and *L. plantarum* preparations with 5-FU significantly reduces HT-29 cell viability and induces apoptosis. The combination also protects normal cells from 5-FU cytotoxicity and increases their viability and survival, demonstrating the dual advantage of probiotic preparations in sensitizing tumor cells to chemotherapy while reducing chemotherapy-induced damage to normal tissues (163). Chemoresistant CRC cells exhibit high metastatic potential, markedly impaired tight-junction integrity, and upregulated claudin-1. Metabolites derived from *Lactobacillus* preparations not only increase the sensitivity of chemoresistant CRC cells to 5-FU but also suppress chemoresistance and metastatic behavior by downregulating *CLDN-1* expression (163) (**Figure 1**; **Table 4A**).

Regarding the alleviation of chemotherapy toxicity, depletion of fecal *Lactobacillus* and *Bifidobacterium* is associated with aggravated oxaliplatin-mediated toxicity, and supplementation with these probiotic preparations can alleviate chemotherapy toxicity. Clinically, peripheral CD45^+^IL-10^+^ cells are downregulated in patients with CRC receiving oxaliplatin, and microbiota-mediated IL-10 production affects chemotherapy tolerance (146). A probiotic preparation of *L. casei* subsp. *rhamnosus* likewise prophylactically alleviates 5-FU/oxaliplatin-induced intestinal damage in CRC models, indicating that specific probiotic strains can preventively reduce chemotherapy-induced intestinal toxicity (164).

#### 8.2.2 Immunomodulation and tissue protection by probiotics in other solid tumors

In non-gastrointestinal solid tumors, probiotics act mainly through systemic immune remodeling and protection of normal tissues. In a urothelial-cancer model, combining *Lactobacillus casei* strain Shirota and *Bifidobacterium breve* with gemcitabine and cisplatin enhanced activation of dendritic cells and granzyme B-positive CD8^+^ T cells and reduced recruitment of Treg cells and cancer-associated fibroblasts (121). This pattern suggests that probiotics can amplify the initial tumor injury caused by cytotoxic drugs by improving antigen presentation, relieving immunosuppression, and reducing the stromal barrier. In breast-cancer models, *Streptococcus thermophilus*, *Lactobacillus bulgaricus*, and their fermented-yogurt preparations alleviated weight loss, intestinal-tissue injury, and treatment-related death without weakening the antitumor effects of 5-FU or capecitabine (133). These preparations therefore support a treatment-tolerance strategy centered on gastrointestinal protection and maintenance of chemotherapy completion (**Figure 4D**; **Table 4A**).

Combination interventions in ovarian cancer show that specific lactobacilli can participate in cisplatin sensitization and intestinal protection. When combined with Tripterygium glycosides, *Lactobacillus paracasei* can affect the Keap1/Nrf2/GPX4 axis, oxidative stress, and ferroptosis-related pathways and enhance the tumor-suppressive effect of cisplatin (165). This combination supports a dual phenotype of cisplatin sensitization and intestinal protection; single-strain intervention, colonization verification, and metabolite-rescue studies would further define the strain-specific contribution.

#### 8.2.3 Reconstruction of microecological functions mediated by prebiotics, synbiotics, and postbiotics

Prebiotics, synbiotics, and postbiotics all aim to restore microbial functions related to chemotherapy efficacy and host tolerance, but their mechanistic bases differ. Prebiotics consist of dietary components that are difficult for the host to digest directly but can be used by specific intestinal bacteria; common types include dietary fiber, inulin, oligosaccharides, and xylan derivatives. Their effects depend on the fermentative capacity of the recipient’s original microbiota and mainly maintain epithelial energy supply, tight junctions, and immune homeostasis by increasing production of SCFAs such as acetate, propionate, and butyrate. When chemotherapy depletes SCFA-producing bacteria, simply increasing the substrate may not restore the target metabolic function; the prebiotic response is therefore markedly recipient dependent (166, 167). Synbiotics combine probiotics with substrates that they can use, thereby increasing the likelihood of survival, colonization, or functional output of the target strains. Postbiotics include inactivated bacterial cells, cell-wall components, extracellular vesicles, bacterial lysates, and purified metabolites and can retain some microbial functions without introducing continuously replicating live bacteria. In patients with neutropenia, impaired mucosal barriers, or intensive chemotherapy, postbiotics can retain selected microbial functions without introducing continuously replicating live bacteria (168) (**Figure 2**; **Table 4A**). Whether this translates into a lower risk of bacteremia or opportunistic infection during intensive chemotherapy requires direct clinical evaluation.

Regarding prebiotic and synbiotic interventions, an *in vitro* colon model showed that prebiotic fiber preparations can counteract 5-FU-induced gut dysbiosis, promote recovery of acetate-producing bacteria such as *Lactobacillus* and *Bifidobacterium*, increase acetate production, and improve 5-FU efficacy and toxicity (169). In a prebiotic–probiotic liposomal co-delivery system, xylan-derivative–probiotic liposomes loaded with 5-FU prolong drug transit and release in the intestine and maintain a higher drug concentration at the tumor site, thereby suppressing tumor growth and improving CRC chemotherapy efficacy (170). The inulin-derivative hybrid liposome loaded with berberine hydrochloride (BLPN) constructed by Xia et al. uses an inulin derivative to prolong berberine retention in the intestine and circulation and produces bidirectional immunomodulatory effects. When BLPN is combined with irinotecan-hydrochloride liposomes (ILPS), it activates innate and adaptive antitumor immune cells, promotes secretion of pro-inflammatory cytokines, and enhances ILPS extravasation in tumor vessels, thereby significantly increasing suppression of CRC growth (171).

Postbiotics achieve barrier protection or direct antitumor effects through nonviable bacterial components with defined functions. Paired samples from patients with gastrointestinal tumors and animal studies show that heat-killed *Bacteroides fragilis* can maintain the epithelial barrier, mitochondrial function, and organoid growth through capsular polysaccharide A and alleviate 5-FU- or irinotecan-associated diarrhea by upregulating BCL2 and downregulating BAX (172). The protective effect is mediated by functional components of the inactivated cells rather than continued colonization by live bacteria, providing a basis for developing quantifiable postbiotic preparations for high-risk patients receiving chemotherapy. Multistrain lysates can also act directly on tumor cells. Preparations such as OxxySlab induce proliferative arrest, cellular senescence, and redox imbalance in bladder-cancer cells and attenuate epithelial–mesenchymal transition, with relatively low toxicity toward normal urothelial cells (173). Lysates contain complex components such as cell-wall constituents, nucleic acids, and metabolites; identifying the active molecules and achieving effective *in vivo* exposure remain key to their further development.

#### 8.2.4 Clinical evidence and implementation considerations

Human trials provide more direct clinical support for selected probiotic formulations, particularly for treatment-toxicity endpoints. In a randomized study of 100 patients with CRC undergoing postoperative chemotherapy, probiotic supplementation reduced gastrointestinal complications, particularly diarrhea, and was accompanied by changes in gut microbial composition and SCFA profiles (152). In a randomized, double-blind, placebo-controlled trial of 99 patients with locally advanced nasopharyngeal carcinoma receiving concurrent chemoradiotherapy, a probiotic combination reduced severe oral mucositis and attenuated treatment-associated reductions in circulating T cell populations (153). A randomized placebo-controlled study in patients with stage I–III breast cancer receiving adjuvant chemotherapy based on pegylated liposomal doxorubicin likewise reported lower incidence and severity of hand–foot syndrome and oral mucositis with probiotic supplementation (29). The published PRO-PLATINUM protocol represents a more chemotherapy-specific prospective design: 124 patients with stage II–IV or platinum-sensitive recurrent high-grade ovarian cancer receiving platinum-based chemotherapy are randomized in a double-blind placebo-controlled design to a defined five-strain probiotic formulation or placebo, with longitudinal gut-microbiome profiling as the primary endpoint and survival, toxicity, metabolic, immune, and additional microbiome measures incorporated as secondary or exploratory endpoints (154). Collectively, these trials position defined probiotic formulations as clinically testable interventions for treatment tolerance and microbiome modulation, while future efficacy-oriented trials can determine whether selected strains also modify tumor-response endpoints (**Table 5**).

Clinical effects of probiotics should be attributed to defined strains, doses, formulations, and treatment settings rather than generalized across a genus or species, because probiotic effects can be strain specific (174). Durable mucosal colonization should be assessed directly. Human multi-omics studies have shown that probiotic mucosal colonization is highly person-, region-, and strain-specific and can produce predominantly individualized and transient effects on the mucosal microbiome (175). Chemotherapy trials should therefore document exact strain composition, viable dose, treatment duration, adherence, concomitant antibiotics, and longitudinal colonization or functional persistence. For live products, manufacturing should define strain identity, purity, viable potency, storage stability, and susceptibility to clinically relevant antibiotics. Particular caution is warranted in patients with severe neutropenia or mucosal-barrier disruption because genomic evidence has demonstrated direct transmission of administered probiotic strains into the bloodstream in critically ill patients (176). Regulatory classification also depends on intended use and jurisdiction: probiotic foods or supplements and live biotherapeutic products intended to treat disease can be subject to different requirements for product characterization, manufacturing quality, clinical evidence, and safety surveillance (158).

### 8.3 Microbiota-targeted phage therapy

Phage therapy exploits the high specificity of phages for their host strains to precisely eliminate pathogenic bacteria involved in chemoresistance while minimizing disruption of other commensal communities. This strategy is suitable for tumors in which the target strain and its resistance-promoting function have been clearly defined and can use lytic phages to weaken microbial drug inactivation, inflammation, and tumor-protective effects (177).

#### 8.3.1 Targeting resistance-promoting bacteria to restore chemotherapy sensitivity

*F. nucleatum* is the most extensively studied cancer- and resistance-promoting bacterium in CRC. It activates β-catenin signaling by binding E-cadherin through the FadA adhesin, maintains autophagy, and blocks ferroptosis, thereby conferring resistance to 5-FU and oxaliplatin in CRC cells. The specific lytic phage FNU1 targeting *F. nucleatum* has been successfully isolated and characterized. The study by Kabwe et al. showed that FNU1 significantly reduced the mass of *F. nucleatum* biofilms and effectively killed bacteria within the biofilm. Functionally, FNU1 completely abolished *F. nucleatum*-induced stimulation of CRC-cell growth, enhanced migratory capacity, autophagy activation, and anti-apoptotic effects. More importantly, FNU1 treatment reversed *F. nucleatum*-mediated chemoresistance and restored tumor-cell sensitivity to chemotherapeutic drugs (178).

With respect to targeting *Bacteroides fragilis*, Ding et al. found by metagenomic sequencing that *B. fragilis* was significantly enriched in chemotherapy nonresponders and was associated with poor survival. The research team established the *B. fragilis*-specific lytic phage VA7, which selectively eliminated *B. fragilis in vivo* without affecting other gut microbiota, restored sensitivity to 5-FU and oxaliplatin, and caused no abnormalities in hepatic or renal function or weight loss during long-term administration (19). With respect to targeting *Escherichia coli*, colibactin-producing pks^+^ *E. coli* produces the genotoxin colibactin through its pks genomic island, induces DNA double-strand breaks in host intestinal epithelial cells, and promotes CRC progression by affecting tumor stemness, treatment resistance, microbial-community remodeling, and immunosuppression (20).

The specific lytic phage Esc-COP-23 targeting pks^+^ *E. coli* significantly alleviates the strong pro-inflammatory transcriptional response, DNA-damage signaling, and epithelial disruption induced by pks^+^ *E. coli* and promotes restoration of intestinal-epithelial junction integrity and repair of DNA double-strand breaks (179). *Bacteroides fragilis* toxin (BFT) secreted by ETBF can cleave E-cadherin, activate the Wnt/β-catenin signaling pathway, and establish a chronic inflammatory microenvironment by upregulating IL-6 and IL-17 and promoting Th17-lymphocyte polarization. In targeting ETBF, ETBF-specific phages significantly alleviated ETBF-induced pro-inflammatory transcriptional responses and epithelial disruption, while transcriptomic analysis showed restoration of the expression of genes related to DNA damage (179).

Regarding elimination of multidrug-resistant bacteria, Fayez et al. isolated multidrug-resistant *E. coli* from feces, evaluated its antibiotic-resistance profile, and established the lytic phage vB_Eco_ZCEC15. The phage showed antibacterial effects under optimized culture conditions and simulated acidic conditions of the human gastrointestinal tract. The study demonstrates phage activity against gastrointestinal MDR *E. coli* under *in vitro* and epithelial-cell conditions; whether such bacterial clearance preserves chemotherapy exposure or efficacy remains to be established (180).

Phage-guided delivery can remain microbiota-targeted when bacterial elimination is an explicit therapeutic component. Zheng et al. developed irinotecan-loaded dextran nanoparticles covalently linked to azide-modified phages to target and suppress *F. nucleatum* growth. In CRC mouse models, this phage–nanoparticle system achieved specific elimination of *F. nucleatum*, and oral or intravenous administration significantly enhanced the efficacy of first-line chemotherapy. In a porcine model, the phage–nanoparticle system had no significant effects on blood-cell counts, immunoglobulins, histamine levels, or hepatic and renal function, indicating a favorable safety profile (181) (**Table 4A**).

#### 8.3.2 Potential microbiota-targeted applications beyond CRC

Infection control may constitute another route through which phages support chemotherapy delivery and treatment completion. In a co-culture system of A549 lung-cancer cells and *Staphylococcus aureus*, Phage K reduced the cell-associated bacterial burden, while individual chemotherapeutic drugs either enhanced or suppressed its antibacterial activity (182). This drug-dependent interaction identifies compatibility between phages and specific chemotherapy regimens as an important translational variable. Clinical studies of infection-directed phage therapy during chemotherapy should therefore pair bacterial-clearance and infection-recurrence endpoints with antibiotic exposure, infection-related hospitalization, chemotherapy delay or dose reduction, relative dose intensity, and oncologic outcomes.

Phage-sensitization strategies in non-gastrointestinal tumors must also distinguish gut-derived microorganisms from microorganisms local to the tumor. Gut microorganisms can regulate treatment responses in distant tumors through circulating microbe-associated molecules, metabolites, the mucosal barrier, and systemic immunity; targeting strategies therefore need to evaluate elimination of the intestinal target bacteria, metabolite changes, and peripheral immune remodeling simultaneously (183). Bacterial communities with cancer-type specificity that are located predominantly within tumor cells or immune cells are present in tumor tissues across multiple cancer types, suggesting that some microbial effects may occur locally within tumors (184); intratumoral bacteria can also metabolize gemcitabine through cytidine deaminase, causing local drug inactivation and reducing effective intratumoral drug activity (9). Before phage therapy, paired fecal, blood, and tumor-tissue analyses should therefore be used to confirm the origin, strain characteristics, spatial distribution, and drug-metabolizing capacity of the target bacteria. For gut-colonizing bacteria, changes in microbial communities, metabolites, and systemic immunity are more suitable as the basis for pharmacodynamic evaluation, whereas targeting intratumoral bacteria requires particular attention to tumor delivery of phages, local bacterial burden, intratumoral drug and metabolite concentrations, and tumor-cell death. Only after a causal relationship between the target bacteria and the resistance phenotype has been validated can the host range, administration route, and efficacy endpoints of the phage be designed with biological interpretability.

Phage specificity can preserve commensal communities, but it also narrows the eligible bacterial hosts and increases the risk of treatment escape. Target strains may alter surface receptors, form protective biofilms, or acquire other resistance mechanisms during therapy. Systemically administered phages can also undergo rapid reticuloendothelial clearance or neutralization by pre-existing and treatment-induced antibodies. Oral delivery must overcome gastric acidity, mucus, and uneven access to tumor-associated bacteria, whereas intratumoral targets require adequate tumor penetration. Chemotherapeutic drugs and antibiotics may further enhance or suppress phage replication depending on the bacterial and drug context. Translation will therefore require isolate-level host-range testing, rational phage cocktails, route-specific pharmacokinetics, serial bacterial-load measurements, and surveillance for phage resistance and neutralizing immunity. Accordingly, oncology-specific evidence for microbiota-targeted phage therapy remains predominantly preclinical, and prospective clinical trials are needed before strain-directed phage strategies can be integrated with cytotoxic chemotherapy (177, 185).

### 8.4 Emerging microbe-inspired adjunctive therapeutic technologies

Microbial structures, tumor tropism, and synthetic-biology principles are increasingly being incorporated into adjunctive anticancer platforms. These approaches extend microbiome research beyond direct ecological intervention by using bacteria, phage-derived structures, bacterial membranes, or microbial components to improve tumor targeting, drug delivery, immune activation, and microenvironmental remodeling. In the context of chemotherapy, their relevance lies primarily in enhancing drug exposure, combining bacterial control with cytotoxic treatment, or integrating chemotherapy into multimodal therapeutic systems.

#### 8.4.1 Engineered microorganisms for tumor-directed adjunctive therapy

Several engineered-microorganism studies have established core enabling functions—tumor-selective sensing and localized therapeutic-payload release—that underpin subsequent combination with systemic anticancer therapy. Lactate-responsive *E. coli* Nissle 1917 can preferentially persist in metabolically defined tumor regions, while photothermally controlled bacterial circuits have been used to trigger local release of cytotoxic proteins (186, 187). Engineered *Salmonella platforms* have delivered immunomodulatory or cytotoxic payloads, including IL-2 and ω-conotoxin MVIIA (123, 188). Saccharomyces-based platforms have likewise been developed for localized delivery of anti-TNF nanobodies and biologics directed at immune checkpoints (54, 189). Collectively, these studies establish the feasibility of tumor-selective microbial sensing and localized delivery of cytotoxic or immunomodulatory payloads, providing an engineering foundation for subsequent combination with systemic anticancer treatment.

Engineered bacteria can also remove physical barriers that limit penetration of chemotherapeutic drugs. Dense hyaluronan and collagen can increase tumor interstitial-fluid pressure, compress microvessels, and restrict drugs such as doxorubicin and gemcitabine from entering poorly perfused regions. After engineered attenuated *Salmonella Typhimurium* secretes HysA hyaluronidase within tumors, it reduces hyaluronan levels and HA–CD44 interactions and enhances tissue penetration and therapeutic effects of doxorubicin or gemcitabine in 4T1 breast-cancer and ASPC-1 pancreatic-cancer models (138). A dual-bacterial system constructed using Nissle 1917 as a chassis can lyse and release ColG collagenase and a CD47 nanobody–PE38KDEL immunotoxin. After collagen is reduced, distribution and tumor-suppressive effects of the immunotoxin and doxorubicin in breast-cancer tissue are both enhanced (139). These studies demonstrate that engineered microorganisms can improve chemotherapy delivery by remodeling physical barriers within the tumor microenvironment, thereby providing a stromal-remodeling strategy for increasing intratumoral drug penetration and therapeutic exposure.

Combined application of engineered microorganisms and platinum-containing chemotherapy has entered early clinical exploration. CRS-207 is an attenuated *Listeria monocytogenes* with deletion of *actA* and *inlB* that expresses human mesothelin. In a phase Ib multicenter study of unresectable malignant pleural mesothelioma, patients received CRS-207 vaccination followed by pemetrexed plus cisplatin and subsequent booster vaccination (140). Among 35 evaluable patients, 1 achieved complete response, 19 partial response, and 10 stable disease, yielding a disease-control rate of 89%; median progression-free and overall survival were 7.5 and 14.7 months, respectively. Paired biopsies showed increased infiltration of CD8^+^ T cells, dendritic cells, and NK cells, an increased CD8^+^ T cell/Treg cell ratio, and a shift of macrophages from an M2-like to an M1-like phenotype (140) (**Table 4B**). This phase Ib combination study establishes the clinical feasibility of combining an engineered microbial therapeutic with pemetrexed–cisplatin and documents accompanying immune remodeling; its single-arm design does not quantify the incremental contribution of CRS-207.

#### 8.4.2 Microbe-inspired biomimetic delivery and combination platforms

Phage-derived materials provide versatile scaffolds for tumor-targeted delivery and combination therapy. EGFR aptamer–siXBP1 nanoparticles constructed on the three-way-junction structure of phage φ29 packaging RNA selectively entered triple-negative breast-cancer cells, reduced XBP1 expression, and enhanced doxorubicin-associated cell killing and tumor suppression *in vitro* and in orthotopic xenografts (137). Related phage-derived or phage-modified platforms have also been developed for tumor imaging, photothermal therapy, and photodynamic treatment (190, 191), illustrating the broader engineering potential of phage-based biomaterials for combination anticancer strategies.

Bacterial membranes can also be repurposed as tumor-directed chemotherapy carriers. Lu et al. used the tumor-associated properties of *F. nucleatum* membranes to construct bacterial membrane nanovesicles functionalized with a PD-L1 antibody (ab-FMNVs). FadA-mediated interaction facilitated tumor-cell targeting and intracellular doxorubicin delivery, resulting in enhanced antitumor activity in primary and recurrent CRC models (192). This strategy illustrates how microbial membrane properties can be harnessed to improve spatially targeted chemotherapy delivery.

Microbe-inspired delivery concepts have also been extended to local chemotherapy. In bladder cancer, the DOX/BCG-PPD nanosystem uses recognition mediated by phenylboronic acid of tumor-cell sialic acid to prolong intravesical retention and increase doxorubicin uptake, while the BCG purified-protein derivative promotes dendritic cell activation and local antigen presentation (141). By integrating local drug retention, immunogenic cell death, and immune activation mediated by microbial components, this platform provides a representative strategy for local chemo-immunotherapy.

Some multifunctional platforms more directly integrate bacterial targeting with chemotherapy. In *F. nucleatum*-associated CRC, MPLO@HA combines metformin, oxaliplatin, lauric acid, and hyaluronic acid to simultaneously reduce the bacterial burden, deliver cytotoxic therapy, and modulate the immunosuppressive tumor microenvironment (193). This design illustrates a bacteria-associated combination strategy in which microbial control and chemotherapy are incorporated into the same therapeutic platform.

In *E. coli*-colonized CRC, Gong et al. developed the IR/CP@BNP@HA platform, which combines the chemotherapeutic drug camptothecin with the photothermal agent IR1048 in a tumor-responsive nanocarrier. Targeting mediated by hyaluronic acid and matrix-responsive degradation facilitate intratumoral penetration and drug release, while treatment also promotes dendritic cell maturation and T cell infiltration (194). This platform demonstrates how chemotherapy can be integrated with bacterial-context targeting, photothermal treatment, and immune activation within a single multimodal system.

Cavalcanti et al. developed a chitosan-coated liposomal system for co-delivery of ciprofloxacin and 5-FU, thereby integrating antibacterial treatment and cytotoxic chemotherapy within a single CRC-oriented delivery strategy (195) (**Table 4B**). Such co-delivery systems provide a practical framework for simultaneously addressing tumor-associated bacterial burden and chemotherapy exposure.

Live bacterial vectors can further integrate chemotherapy with immune and physical treatment modalities while simultaneously influencing host microbial ecology. Jiang et al. used the tumor tropism and hypoxia-targeting properties of *Magnetospirillum gryphiswaldense* to construct MSR-CPT/APPs, a magnetotactic bacterial platform carrying camptothecin and an anti-PD-L1 peptide for targeted chemo–magnetothermal immunotherapy of CRC (196). The treatment induced DNA damage and cGAS–STING-associated immunogenic signaling, promoted M1-like macrophage polarization and sustained T cell recruitment, and reduced immunosuppressive-cell populations. In parallel, MSR-CPT/APPs increased the relative abundances of commensal taxa including Lachnospiraceae and *Alistipes*, increased SCFAs and citrulline, and reduced L-glutamine and kynurenic acid. These concurrent tumor, immune, and microbial effects illustrate the potential of live microbe-inspired platforms to integrate chemotherapy delivery with systemic immune and metabolic remodeling in multimodal cancer treatment (196) (**Tables 4A,B**).

Engineered living vectors and nonliving microbe-inspired carriers have distinct risk profiles. Live vectors may be rapidly cleared, trigger host immunity, colonize off-target tissues, or cause bacteremia in patients with impaired barriers. Genetic instability, payload loss, horizontal gene transfer, and failure of biological-containment systems must also be considered. Clinical development therefore requires validated kill switches, genetic-stability testing, manufacturing release criteria, organ-burden and shedding measurements, and patient selection based on immune status (197). Repeated dosing may be limited by adaptive immunity or altered biodistribution. Nonliving bacterial membranes and vesicles avoid replication but still require control of endotoxin content, cargo loading, batch consistency, off-target inflammation, and long-term clearance. These requirements should be evaluated alongside antitumor efficacy before microbe-inspired platforms are considered clinically feasible adjuncts to chemotherapy or other anticancer modalities.

The translational requirements of microbiome-related strategies depend on both the microbial compartment and the intervention class. Approaches targeting the gut microbiota can influence chemotherapy through microbial drug metabolism, barrier function, systemic immunity, and treatment tolerance, whereas strategies directed at local or intratumoral microorganisms act within organ-specific microbial niches. Emerging microbe-inspired technologies provide an additional translational route through tumor-directed drug delivery, stromal remodeling, immune activation, and multimodal combination treatment. Clinical evaluation should therefore align efficacy, pharmacodynamic, and safety endpoints with the principal mechanism of each strategy (**Tables 4A,B**).

### 9.1 Current challenges

For strategies targeting the gut microbiota, major challenges include limited clinical evidence, incomplete causal attribution, inconsistent technical standards, and uncertainty regarding reproducibility across cancer types and treatment regimens. Evidence maturity is also highly uneven across tumor types: CRC currently provides the most integrated clinical and mechanistic evidence, whereas several extraintestinal cancers remain supported mainly by small observational cohorts or preclinical models. Existing human studies are predominantly observational cohorts with limited sample sizes and substantial variation in cancer type, disease stage, treatment regimen, efficacy definition, and sampling time point. A 2025 systematic review included only 22 human studies that simultaneously assessed the fecal microbiota and chemotherapy response, efficacy, or toxicity, and most candidate bacteria had not shown consistent associations across centers and populations (3). Chemotherapy-associated microecological features are therefore more likely to be specific to cancer type and treatment regimen, and a single genus is unlikely to serve as a universal predictive indicator. At the same time, genus- or species-level abundance is insufficient to reflect the microbial functions that actually determine drug response. Different strains of the same species can produce opposing effects because of differences in drug-metabolizing enzymes, virulence islands, biofilm formation, and immunologically active components (7). Detection of relevant functional genes by metagenomics also does not prove that they are continuously expressed during treatment and generate sufficient metabolites (198). Microbiome research therefore needs to advance beyond taxonomic associations to the levels of strains, functional genes, and active metabolites (**Tables 4A,B**).

Temporal sequence and control of confounding factors remain major weaknesses in causal identification. Chemotherapy itself can alter dietary intake, intestinal-luminal substrates, mucosal permeability, and supportive medications. Microbial changes observed after treatment may therefore participate in acquired resistance, but may also merely result from drug exposure, disease progression, or altered nutritional status. Region, diet, age, body weight, hepatic and renal function, and medications such as antibiotics, proton-pump inhibitors, glucocorticoids, and opioids can also markedly influence the baseline microbiota. If these variables are unevenly distributed among efficacy groups, a predictive model may identify differences in center, medication, or lifestyle rather than chemosensitivity (124, 199). Inconsistency in detection and analysis procedures further limits reproducibility. The 16S rRNA amplification region, DNA-extraction method, sequencing depth, reference database, and bioinformatics pipeline can all alter the resulting differential taxa (200). Low-biomass samples such as tumor tissue, blood, and urine are particularly susceptible to reagent and environmental contamination, making appropriate contamination controls essential for interpretation (201).

Although animal and *in vitro* models have demonstrated that some microbial effects are modifiable, changes in microbial-community structure do not equate to restoration of function. Increased microbial diversity, colonization by donor bacteria, or reduction of target bacteria must each be further linked to metabolite output, immune changes, drug exposure, and tumor response (202). Germ-free mice, antibiotic-treated models, immunodeficient mice, and patient-derived xenograft models each have limitations, and no single model can fully reproduce the heterogeneity of patient microbiota, immunity, and tumors (203, 204). Therefore, changes in microbial composition or tumor suppression in a single model alone remain insufficient to demonstrate that microbial function has been restored or to predict the actual efficacy of the same intervention in different patients.

Clinical implementation barriers are intervention specific. For FMT, reproducibility and safety depend on rigorous donor qualification, screening for enteric pathogens and multidrug-resistant organisms, standardized collection and processing, lot-level traceability, engraftment monitoring, and recipient-specific infection surveillance (155, 157). Donor-derived extended-spectrum β-lactamase-producing *Escherichia coli* bacteremia, including a fatal case, demonstrates the potential consequences of inadequate transmission-risk control (156). For probiotics, efficacy should be attributed to defined strains, doses, and formulations rather than broad taxonomic groups (174), while colonization and functional persistence require longitudinal verification because mucosal engraftment can be highly individualized and transient (175). Live probiotic preparations also warrant particular caution in patients with impaired host defenses because direct transmission of probiotic strains into the bloodstream has been demonstrated by genomic analysis (176). Phage products require isolate-level host-range confirmation, reproducible potency, and surveillance for phage resistance and host neutralizing immunity (177, 185). Engineered bacteria introduce additional requirements for genetic stability, validated biocontainment or kill-switch systems, monitoring of shedding and off-target colonization, and consistent manufacturing and release testing (197). Regulatory requirements are not uniform across microbiome-directed products and depend on intended use, product classification, and jurisdiction; clinical development should therefore prospectively define product identity, manufacturing specifications, release criteria, traceability, biosafety monitoring, and adverse-event reporting according to the biological properties and risk profile of each intervention (158).

### 9.2 Future directions

Future research should move from descriptive microbial associations toward strain-level functional validation and longitudinal causal analysis. Clinical studies need to perform serial sampling before and during treatment and simultaneously record key clinical variables. Multi-omics technologies, including metagenomics, metatranscriptomics, and metabolomics, can help resolve the effects of microorganisms on chemotherapy response at a functional level, and their combination with drug and metabolite measurements can distinguish microbial changes from actual pharmacokinetic effects. On this basis, causal validation must establish a chain of evidence from microbial exposure to clinical outcome. Strategies such as strain reintroduction, functional-gene manipulation, and metabolite supplementation can be used to identify key mechanistic nodes. Combining patient-derived organoids, animal models, and clinical samples can improve the generalizability of the results.

Microbial biomarkers require external validation in independent multicenter cohorts and must demonstrate incremental predictive value beyond existing clinical indicators. Cross-cohort studies show that some microbial features have a degree of reproducibility but also substantial population differences, indicating that single-center results cannot be directly generalized. Predictive models should follow standardized reporting guidelines such as TRIPOD+AI to improve the completeness, transparency, and interpretability of model reporting (205). Future clinical trials should also use mechanism-based stratified designs and enroll patients with specific abnormalities in microbial function into the corresponding interventions. Different intervention strategies, such as phages, enzyme inhibitors, defined microbial consortia, or metabolite supplementation, should be matched to their mechanisms of action rather than selected empirically on the basis of changes in microbial abundance alone.

The development of intervention products should move from whole-ecology replacement toward functionally defined preparations with controllable potency. Defined microbial consortia, postbiotics, and metabolite preparations can improve controllability and reduce some risks. Phages can provide strain-specific targeting of resistance-associated microorganisms, whereas engineered bacteria can enable programmable tumor localization and controlled therapeutic-payload release. Product selection should be individualized according to the patient’s immune status and disease characteristics. Corresponding quality-control systems for these products should cover strain identity, functional potency, and safety-risk assessment. FMT and defined microbial consortia require strain-level tracking systems and source analysis of potential infection events. Relationships among dose, exposure, and effect should also be defined, and long-term ecological effects should be evaluated.

Clinical evaluation should be aligned with the mechanism of the intervention and simultaneously consider tumor control, treatment toxicity, and changes in microbial function. Changes in microbial communities or metabolites can serve as mechanistic indicators, but they must be consistent with clinical benefit to support therapeutic value. Research procedures should also be standardized according to guidelines such as STORMS, including sample collection, experimental procedures, and data analysis, to improve reproducibility and comparability (206). Multicenter studies in particular require unified operating procedures, negative and positive controls, and control of center and batch effects to reduce systematic bias.

For strategies targeting the gut microbiota, future priorities include identifying strain-level functions with reproducible effects, establishing temporal causal relationships between microbial changes and resistance or toxicity, and developing interventions with defined composition, stable potency, and monitorable ecological risks. For microbe-inspired therapeutic technologies, priorities include reproducible tumor targeting, controlled payload release, manufacturing consistency, biosafety, and integration with standard chemotherapy. Clinical translation of either strategy will ultimately require mechanism-matched evidence of improved tumor control, treatment tolerance, drug exposure, or other clinically relevant outcomes.

## Glossary

5-FU: 5-fluorouracil
ACE: abundance-based coverage estimator
AHR: aryl hydrocarbon receptor
AKT: protein kinase B
ALDH: aldehyde dehydrogenase
AOM/DSS: azoxymethane/dextran sodium sulfate
AP-1: activator protein 1
AUC: area under the curve
BAX: BCL2-associated X protein
BCG-PPD: Bacillus Calmette–Guérin purified protein derivative
BCL2: B-cell lymphoma 2
BFT: Bacteroides fragilis toxin
BLPN: berberine-loaded inulin-derivative hybrid liposome
cGAS–STING: cyclic GMP–AMP synthase–stimulator of interferon genes
CI: confidence interval
cIAP2: cellular inhibitor of apoptosis protein 2
CLDN1: claudin 1
CoPEC: colibactin-producing Escherichia coli
CRC: colorectal cancer
CSC: cancer stem cell
DC: dendritic cell
DCA: deoxycholic acid
DOX: doxorubicin
EGFR: epidermal growth factor receptor
EMT: epithelial–mesenchymal transition
ER: endoplasmic reticulum
ETBF: enterotoxigenic Bacteroides fragilis
ETEC: enterotoxigenic Escherichia coli
FadA: Fusobacterium adhesin A
Fas: Fas cell surface death receptor
FMT: fecal microbiota transplantation
FOLFOX: folinic acid 5-fluorouracil and oxaliplatin
GABA: γ-aminobutyric acid
GLUT: glucose transporter
GPX4: glutathione peroxidase 4
GSDME: gasdermin E
HA: hyaluronic acid
HDAC: histone deacetylase
HFD: high-fat diet
HR: hazard ratio
IC50: half-maximal inhibitory concentration
IDO: indoleamine 2,3-dioxygenase
IFN-γ: interferon γ
IL: interleukin
ILPS: irinotecan hydrochloride liposomes
IPA: indole-3-propionic acid
LASSO: least absolute shrinkage and selection operator
LCA: lithocholic acid
LEfSe: linear discriminant analysis effect size
LPS: lipopolysaccharide
mCRC: metastatic colorectal cancer
MCT: monocarboxylate transporter
MDSC: myeloid-derived suppressor cell
MIF: macrophage migration inhibitory factor
MMP: matrix metalloproteinase
mTOR: mammalian target of rapamycin
nCRT: neoadjuvant chemoradiotherapy
NF-κB: nuclear factor kappa B
NK: natural killer
NKG2A: natural killer group 2A
NLRP3: NLR family pyrin domain containing 3
NSCLC: non-small cell lung cancer
OR: odds ratio
OS: overall survival
PCoA: principal coordinate analysis
PD-1: programmed cell death protein 1
PDK2: pyruvate dehydrogenase kinase 2
PD-L1: programmed death-ligand 1
PDX: patient-derived xenograft
PFS: progression-free survival
PI3K: phosphoinositide 3-kinase
RAS: rat sarcoma
RECIST: Response Evaluation Criteria in Solid Tumors
ROS: reactive oxygen species
sCD14: soluble cluster of differentiation 14
SCFAs: short-chain fatty acids
SMCT1: sodium-coupled monocarboxylate transporter 1
sRAGE: soluble receptor for advanced glycation end products
STAT3: signal transducer and activator of transcription 3
T3SS: type III secretion system
TCGA: The Cancer Genome Atlas
TGF-β: transforming growth factor β
TIGIT: T cell immunoreceptor with immunoglobulin and ITIM domains
TIM-3: T cell immunoglobulin and mucin domain-containing 3
TLR: Toll-like receptor
TME: tumor microenvironment
TNBC: triple-negative breast cancer
TNF-α: tumor necrosis factor α
Treg: regulatory T cell
UPLC–MS/MS: ultra-performance liquid chromatography–tandem mass spectrometry
VEGF: vascular endothelial growth factor
XBP1: X-box binding protein 1
YAP: Yes-associated protein
ZO-1: zonula occludens 1
